# Reversible encapsulations and stimuli-responsive biological delivery from a dynamically assembled cucurbit[7]uril host and nanoparticle guest scaffold†

**DOI:** 10.1039/c8tb01596a

**Published:** 2018-09-25

**Authors:** Santu Sinha, Nilanjana Das Saha, Ranjan Sasmal, Divyesh Joshi, Soumya Chandrasekhar, Monica Swetha Bosco, Sarit S. Agasti

**Affiliations:** a New Chemistry Unit, Jawaharlal Nehru Centre for Advanced Scientific Research (JNCASR), Bangalore, Karnataka 560064, India; b Chemistry & Physics of Materials Unit, Jawaharlal Nehru Centre for Advanced Scientific Research (JNCASR), Bangalore, Karnataka 560064, India; c Molecular Biology and Genetics Unit, Jawaharlal Nehru Centre for Advanced Scientific Research (JNCASR), Bangalore, Karnataka 560064, India; d School of Advanced Materials (SAMat), Jawaharlal Nehru Centre for Advanced Scientific Research (JNCASR), Bangalore, Karnataka 560064, India

## Abstract

The positive outcome of any therapeutic molecule requires control over its delivery rate. When delivered without control, administration of large doses is required to stimulate a therapeutic effect, frequently leading to increased toxicity or undesirable side effects. Recent advances introduced “smart” materials that actively release drugs in response to environmental stimuli. Although a variety of endogenous and exogenous triggers are reported, they are either difficult to control or lack tissue penetration depth. We report here a dynamic drug delivery scaffold based on a cucurbit[7]uril (CB[7]) host and benzylammonium functionalized gold nanoparticle (AuNP) guest that utilizes a bioorthogonal small molecule to achieve therapeutic control. In addition to their ability to reach deep tissue, small molecule activation is benefitted by their external controllability. Through cell culture studies we demonstrate that the host–guest supramolecular scaffold provides a nontoxic platform that effectively encapsulates a variety of therapeutic molecules and controls the payload release upon exposure to a high-affinity competitive guest molecule. This study presents a new strategy for controlling drug release rate through the use of competitive interactions of orthogonally presented guest molecules with immediate advantages in dosage control.

## Introduction

The outcome of any therapeutic intervention is directly coupled to the way in which drug molecules or pharmacologically active agents are administered.^1,2^ The administration process controls various important parameters, including pharmacokinetics, drug metabolism and toxicity. The systemic delivery that has been in practice since the inception of drugs is traditionally administered either by an oral or an intravenous route. This standard administration process typically leads to a peak-valley plasma concentration–time profile, leading to adverse effects, especially of a drug with small therapeutic index. In order to maintain drug concentration within the therapeutic window, drug delivery systems (DDSs) were developed.^3,4^ DDSs were engineered to deliver drugs in response to a stimulus leading to regulated and on-demand drug presentation.^5–8^ Various endogenous approaches (*e.g.*, pH, redox and enzyme) that rely on a specific physicochemical characteristic of the tissue microenvironment were developed.^9,10^ However, the endogenous triggers are difficult to control due to their tremendous variation from one patient to another.^11^ In addition, for many cases, the target site lacks overexpression of suitable endogenous triggers.^5^ Alternatively, controlled delivery systems that rely on exogenous stimuli, such as light, ultrasound, magnetic field, and temperature, were also employed.^12^ Although these exogenous stimuli are easier to control, they lack tissue penetration depth.^5^ In light of these challenges, we set out to develop a delivery system that makes use of a bioorthogonal small molecule to achieve therapeutic control as such stimulus can be controlled externally and has the ability to reach deep tissue.^13^

**Scheme 1:**
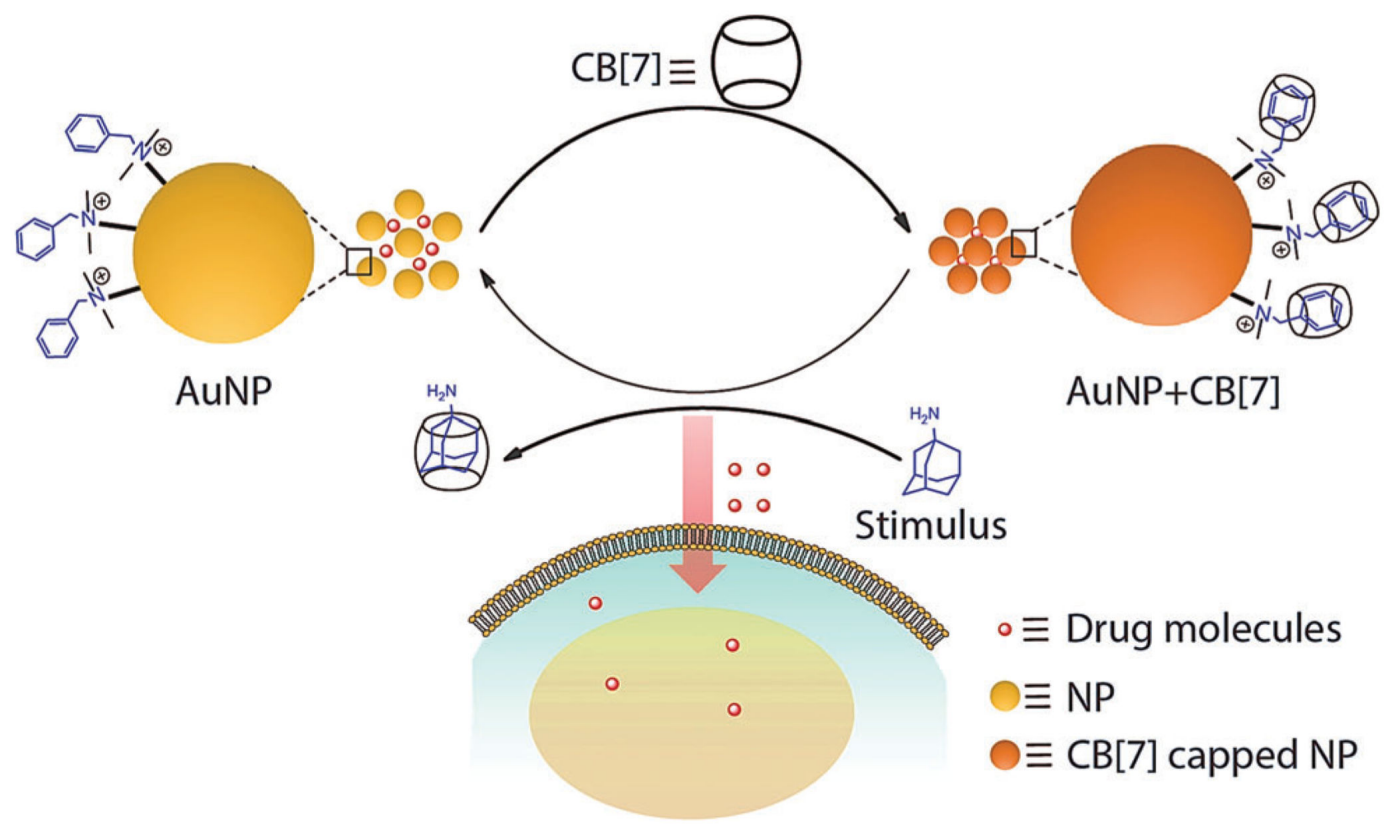
Dynamic nanoparticle assembly and design of a small molecule stimuli responsive drug delivery system. Assembly of AuNPs driven by CB[7] and disassembly by ADA due to host–guest complexation of CB[7] with ADA. Triggered released of drug molecules from assembly and uptake by the cell.

Supramolecular host–guest chemistry facilitates programmable and controllable engineering of molecular systems through the incorporation of various non-covalent molecular recognition motifs.^14–16^ As they employ weak and reversible non-covalent interactions for their recognition process, supramolecular systems can be engineered to assemble and disassemble spontaneously in response to a range of triggers, including presentation of complimentary guest molecules.^17–20^ In this communication, we report a dynamic drug delivery scaffold based on a CB[7] host and benzylammonium functionalized AuNP guest with programmed response towards a bioorthogonal small guest molecule (Scheme 1). Among various non-covalent host–guest building blocks, a CB[7] based synthetic receptor was specifically selected for this study due to its high levels of affinity and chemoselectivity, particularly for recognition processes in a biological context.^21–32^ Host–guest recognition between the AuNP guest and CB[7] host forms a non-toxic assembly that is capable of encapsulating drugs as well as various macromolecular therapeutic agents in an efficient manner. This host–guest complex can be disassembled upon presentation of a high affinity competitive guest molecule, such as adamantylamine (ADA). The disassembly process results in triggered release of therapeutic molecules, thereby inducing cell death. We show the ADA concentration dependent change in cell toxicity, effectively correlating the trigger amount with the dose of the released drug. We expect that this study will open up new opportunities for regulating therapeutic systems through the use small molecule triggers, with immediate advantages over dosage control and improved therapeutic effect.

## Results and discussion

We designed the guest scaffold by using AuNPs with a core diameter of ~2 nm and ~5 nm. 2 nm AuNPs were prepared *via* the Brust Schiffrin method whereas a heat induced size evolution method was adopted for preparing 5 nm particles. We used a place exchange reaction to decorate AuNPs with benzyl-ammonium functionalities, which serve as a guest moiety for recognition by CB[7]. Matrix Assisted Laser Desorption/Ionization-Mass Spectrometry (MALDI-MS) was used to verify the chemical functionality on the NP surface (Fig. S3, ESI†). Benzylammonium functionalized AuNPs were found to be soluble in water. Transmission electron microscopy (TEM) characterization and Dynamic Light Scattering (DLS) measurement showed that the functionalized AuNPs exist as discrete NPs in solution (Fig. S1 and S4, ESI†).

**Fig. 1:**
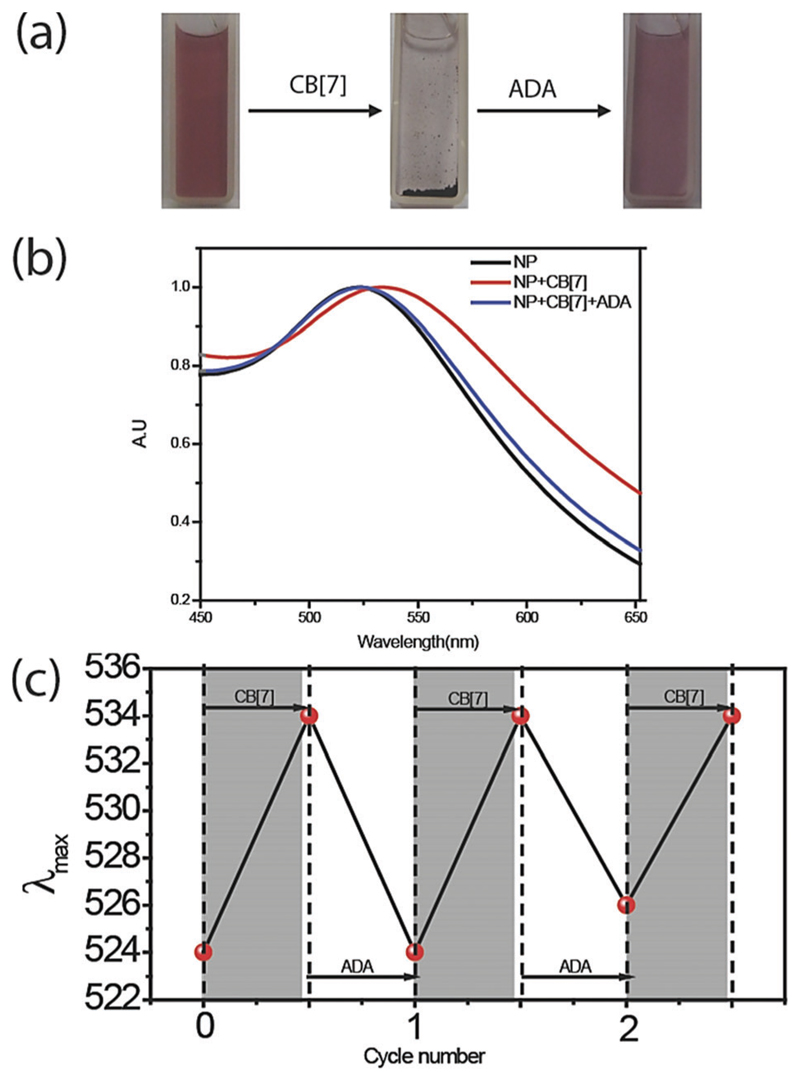
(a) Reversible assembly of AuNPs. Assembly driven by supramolecular interaction between AuNP and CB[7], and disassembly driven by high affinity guest ADA. 200 nM AuNPs were used to form the assemblies. (b) UV-Vis spectroscopy study of the NP assembly and disassembly process. NP plasmon absorption maximum shifted to the right side in the case of NP + CB[7] and the absorption maximum again came back to the initial stage in the case of NP + CB[7] + ADA. A relatively lower concentration (37 nM) of AuNP was used for this study to achieve smaller and dispersed assembly. (c) Reversible changes in absorption maximum of 5 nm AuNPs by alternative addition of CB[7] and ADA.

We used UV-Vis spectroscopy to study the effect of the CB[7] host on the behavior of the NP guest. Monitoring the surface plasmon absorption band is an extremely powerful and sensitive technique to predict the proximity between AuNPs. We observed that surface functionalized NPs were stable in solution for months and the presence of a surface plasmon band was observed at 525 nm for 5 nm AuNPs. However, upon addition of CB[7] into the benzylammonium functionalized AuNP solution, we observed an immediate red shift of the surface plasmon band to 534 nm, indicating formation of AuNP assemblies (Fig. 1a and b). We believe that CB[7] capping of NPs leading to extended NP assembly is an effect of strong interaction between peripheral CB[7] molecules, as observed previously by Kim *et al.* in the case of CB[7] gel formation.^33^ To validate that the assembly is driven *via* host–guest mediated interaction between the synthetic receptor, CB[7], and the benzylammonium ligand on the AuNP surface, we used a competitive guest to remove the CB[7] host from the NP periphery with an expectation to reverse the assemblies to a dispersed state. For this purpose, the self-assembled AuNPs were treated with ADA guest which possess much higher affinity towards CB[7] (*K*_a_ = 1.7 × 10^12^ M^−1^) as compared to the benzylammonium guest (*K*_a_ = 1.6 × 10^5^ M^−1^).^34^ We observed that addition of ADA triggered the AuNPs to disassemble with concomitant blue shift in the plasmon band to 525 nm, demonstrating the importance of host–guest chemistry for assembly formation. Additionally, we found that the reversible assembly and disassembly process can be repeated for multiple cycles by successive addition of CB[7] and ADA (Fig. 1c), indicating the robust nature of the supramolecular system. Reversible transition of the system was also proved by reversible changes in the size of the NP solution *via* dynamic light scattering (DLS) studies (Fig. S5, ESI†). To ensure the formation of stable and robust assemblies, the same were subjected to higher temperature and sonication. It was observed that the integrity of the assemblies was well maintained even under these adverse conditions (Fig. S6, ESI†).

**Fig. 2:**
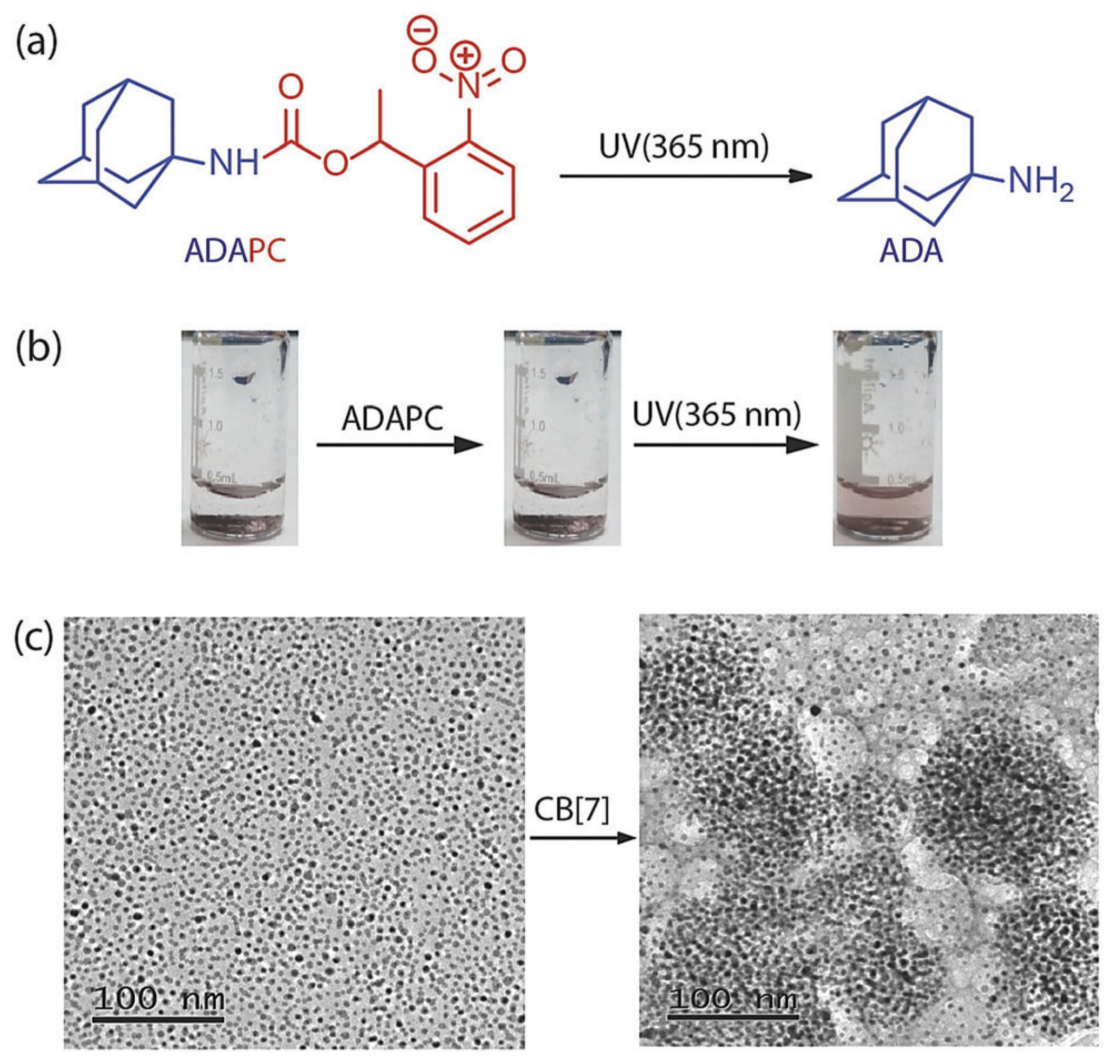
(a) Photocleavage of ADAPC to ADA. (b) Images of light triggered disassembly of an NP-CB[7] complex. An assembly treated with ADAPC does not disperse but irradiation with UV light disassembled the assembly and generates dispersed nanoparticles. (c) TEM images of 5 nm AuNPs in a dispersed state (left) and an aggregated state (right).

The specificity of the assembly towards the ADA trigger was confirmed by employing a photocaged ADA construct (ADAPC, Fig. 2a). In this construct, a photocleavable nitrobenzyl group was attached with the available amine functionality of the ADA moiety to significantly reduce the affinity of ADA towards CB[7]. We observed that incubation of the assemblies with a photo-caged version of ADA did not result in any disassembly process, indicating the role of the high-affinity competitive guest to trigger the disassembly process. However, a brief illumination of the sample with 365 nm light resulted in uncaging of ADAPC, leading to the release of ADA and dispersion of NPs (Fig. 2b). Finally, the CB[7] mediated NP assembly was subjected to morphological analysis using TEM. As shown in Fig. 2c, TEM images exhibited a clear difference between the dispersed state and CB[7] mediated assembled state of the NPs. The assembled state showed the presence of spherical aggregates in the TEM images. In addition, to achieve smaller and solution dispersed assembly from our system, we have varied the ratio between AuNPs and CB[7]. We observed that a ration of 1 : 100 between AuNPs and CB[7] resulted in the formation of dispersed assemblies with a dimension of ~148 nm. These assemblies were characterized *via* DLS as well as AFM (Fig. S8, ESI†).

CB[7] mediated *in situ* formation of supramolecular assemblies presents an exciting opportunity for therapeutic encapsulation. We envisioned that during the formation of this supramolecular assembly there would be a generation of void spaces inside the scaffold and these spaces can be effectively utilized to trap a wide range of therapeutically active molecules. First, we used doxorubicin (DOX) as a model system to test the ability of these assemblies to trap small drug molecules. DOX was an ideal choice for this study due to its inherent fluorescence property that helps in quantification of the encapsulation efficiency. For encapsulation studies, AuNPs were first added into a DOX solution with an NP : DOX ratio of 1 : 10. Subsequently, we formed NP assemblies *via* addition of CB[7]. The assemblies were allowed to settle for a few minutes and the supernatant was collected for quantifying DOX *via* fluorescence measurement. The fluorescence intensity of the supernatant solution was significantly lower when compared against the fluorescence intensity of the incubated solution, indicating successful encapsulation of drug molecules (Fig. S9, ESI†). The encapsulation efficiency was estimated to be ~51%. In addition to DOX, we have also tested other drug molecules, like camptothecin (Fig. S10, ESI†). In all these cases the assemblies showed abilities to encapsulate the drug molecules irrespective of their diverse structural features. Besides drug molecules, the abilities to encapsulate larger biomolecules were also tested. We observed that the assemblies are capable of encapsulating small DNA molecules as well as proteins (Fig. S11, ESI†). Importantly, we also observed that the assembled system can encapsulate enzymes in its active and functional state. For visual demonstration of enzyme encapsulation and catalysis, b-galactosidase was encapsulated into the assembly. We looked at multiple cycles of enzyme activity of the NP assembly using an *o*-nitrophenyl-β-galactoside substrate. During each cycle, after a few minutes of incubation with the substrate, the clear solution turned to yellow, confirming the retention of activity of the encapsulated enzymes (Fig. S12, ESI†). Overall, these results indicate the abilities of these assemblies to act as a versatile platform for encapsulation of various biologically active molecules with retention of their activity.

**Fig. 3:**
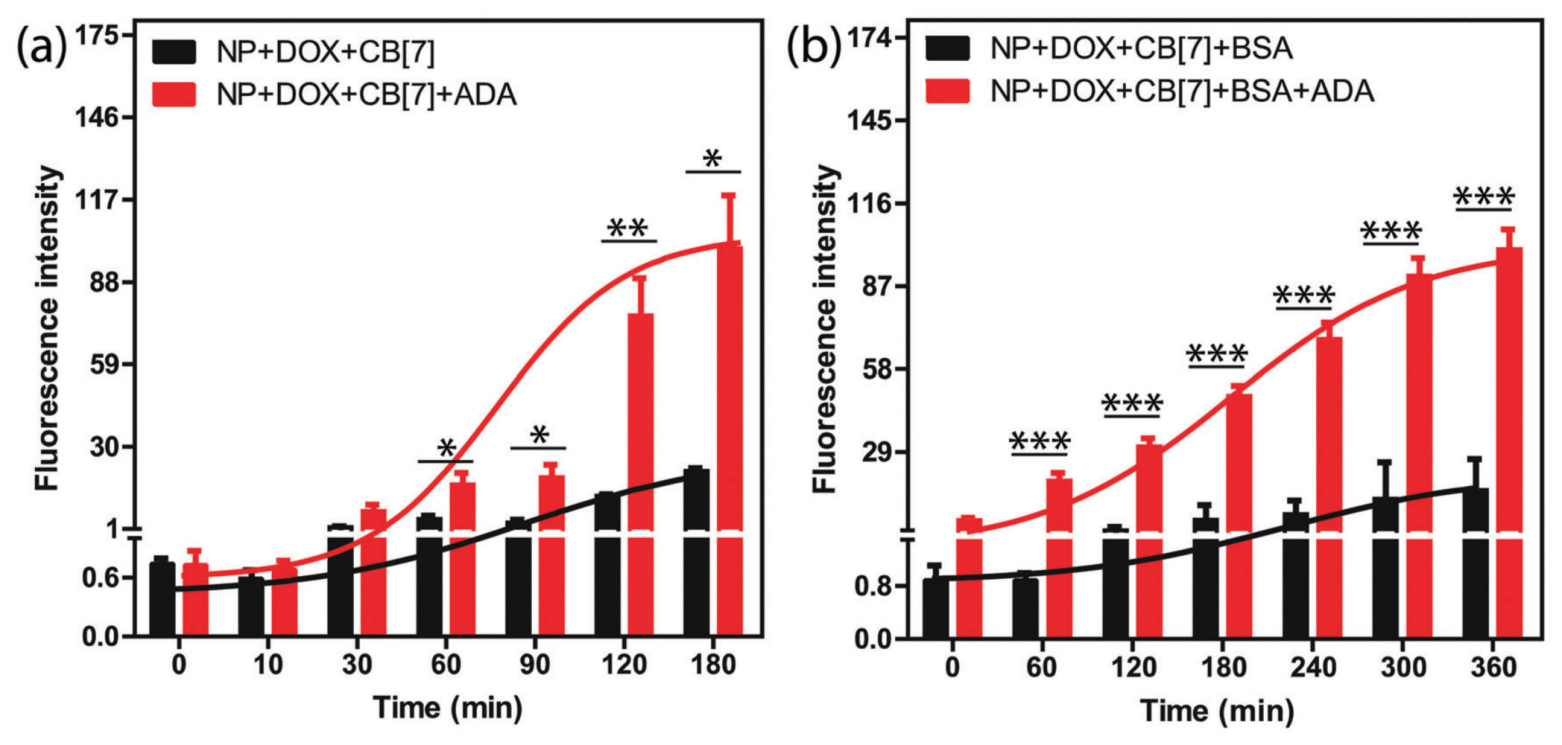
Drug release profile of DOX from the AuNP assembly. (a) Comparison of the DOX release profile from assembly only and assembly triggered by ADA. (b) DOX release profile after coating the assemblies with BSA. The lines were drawn to show the release trend. The studies were performed in triplicate and the error bars represent standard error of the mean. * *p* ≤ 0.05, ** *p* ≤ 0.01, *** *p* ≤ 0.001.

We tested the stimuli responsive release of drug molecules from the supramolecular assemblies using an implant-mimicking device where we used DOX as a model drug molecule. An implant-mimicking device was prepared to separate drug encapsulated nanoparticle assemblies from the released drug molecules. A nitrocellulose membrane (pore diameter of 0.2 μm) was used as a barrier membrane through which drug molecules can easily diffuse out while keeping the NP assemblies inside the device (Fig. S13, ESI†). After formation of the DOX containing supramolecular assembly, it was transferred into the device, which was then placed inside the wells of a 24-well plate containing cell culture medium. Two sets of experiments were performed; in one case a release study was performed only from the assembly and in other case, release was triggered *via* addition of ADA into the assembly. Fluorescence measurement of the culture medium clearly demonstrates an increased release rate for the ADA triggered system as compared to the assembly itself (Fig. 3a). This is due to the triggering of the disassembly process by ADA that leads to better escape probability for the trapped drugs. The released drug amount in normal diffusion mode was ~19% whereas ~72% drug was released when triggered with ADA. Importantly, we observed that the amount of released drug could be easily controlled by the amount of given trigger (*i.e.* ADA), indicating the ability to externally control the drug-dosing amount (Fig. S14, ESI†). In addition, we tested the release kinetics of these assemblies after coating the assemblies with bovine serum albumin (BSA). As we expect that these assemblies will adsorb serum proteins upon presentation to the biological environment, this experiment simulates the release kinetics in an *in vivo* scenario. We observed a significantly increased release rate for the ADA triggered assemblies as compared to the normal diffusion controlled release for the non-triggered assemblies (Fig. 3b). We also show that parameters like CB[7] amount and AuNP size can be further tuned to achieve better control over drug release *via* utilization of a low concentration of ADA trigger (Fig. S15 and S16, ESI†). Next, we tested whether the release profile is selective towards the ADA trigger. For this study, ADAPC was used as a control, where it was added into the assembly to test the drug release profile. A release rate which is increased upon light irradiation indicated the specificity of the ADA trigger (Fig. S17, ESI†). This study also indicates that this supramolecular host–guest system can be potentially designed to respond to a versatile trigger.

**Fig. 4:**
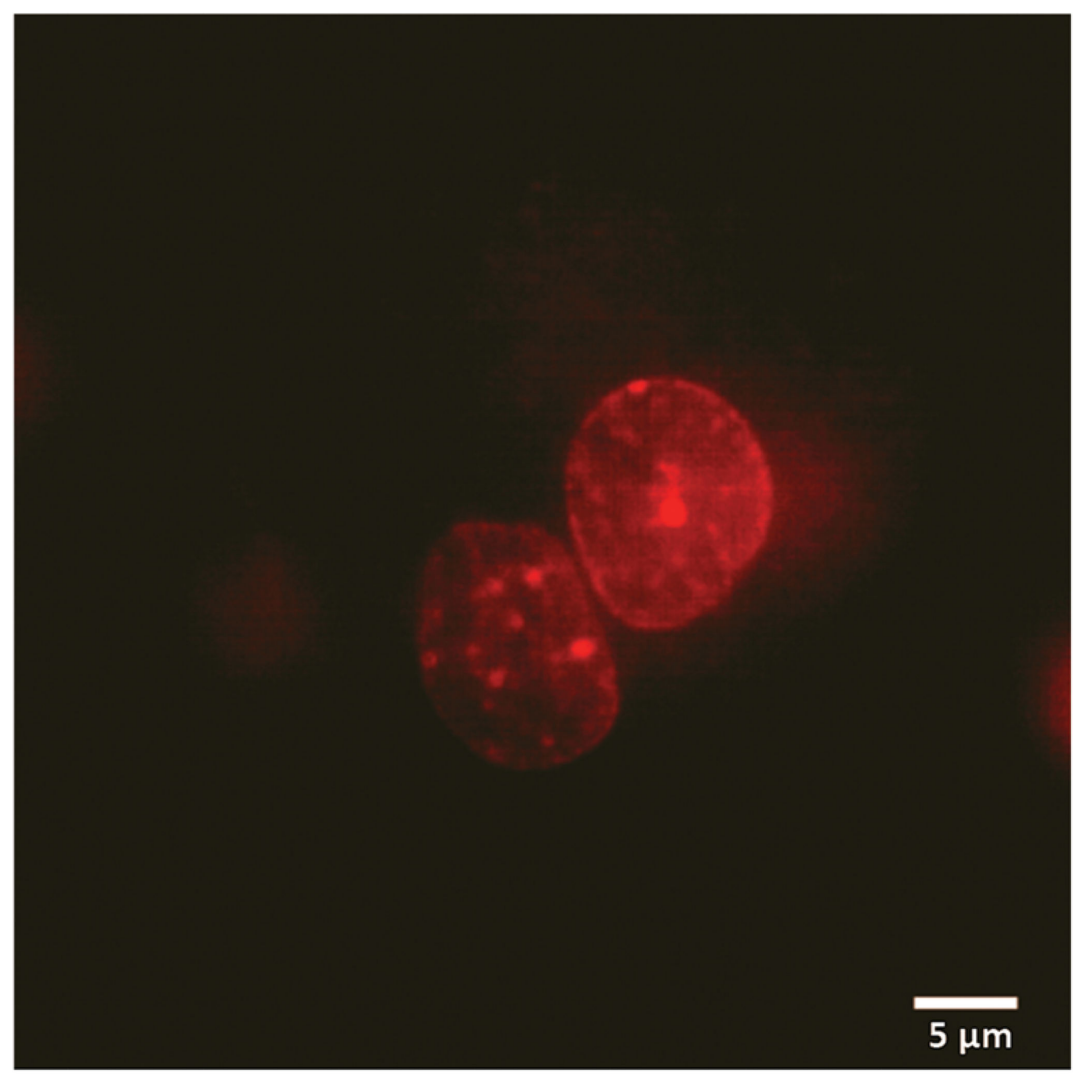
Fluorescence image of the nucleus of Hela cells treated with NP + DOX + CB[7] and triggered with ADA for 6 h. Assembly encapsulated DOX was released after the triggered action of ADA and enters into the cellular nucleus.

We used fluorescence microscopy to demonstrate intra-cellular delivery of the payload from the host–guest supramolecular assembly. HeLa cells were placed on a glass bottom 35 mm imaging dish. The assembly containing DOX and ADA was placed inside the imaging dish using the implant-mimicking device. Cells were kept inside an incubator for 6 h. An imaging experiment was performed after removing the implant and subsequently washing the cells with PBS. Intense DOX fluorescence was observed from the nuclear region of the cell when excited with a 561 nm laser, indicating successful delivery of the payload to its target site (Fig. 4). Additionally, we have performed an imaging study with a control group where a DOX containing AuNP assembly was employed without an ADA trigger. As shown in Fig. S18 (ESI†), microscopy images denoted significantly enhanced fluorescence from the cell nucleus in the presence of ADA. Intensity profile analysis showed >3-fold increase of fluorescence for the ADA trigger as compared to the control group, indicating an enhanced amount of DOX delivery upon triggered activation (Fig. S18, ESI†).

**Fig. 5:**
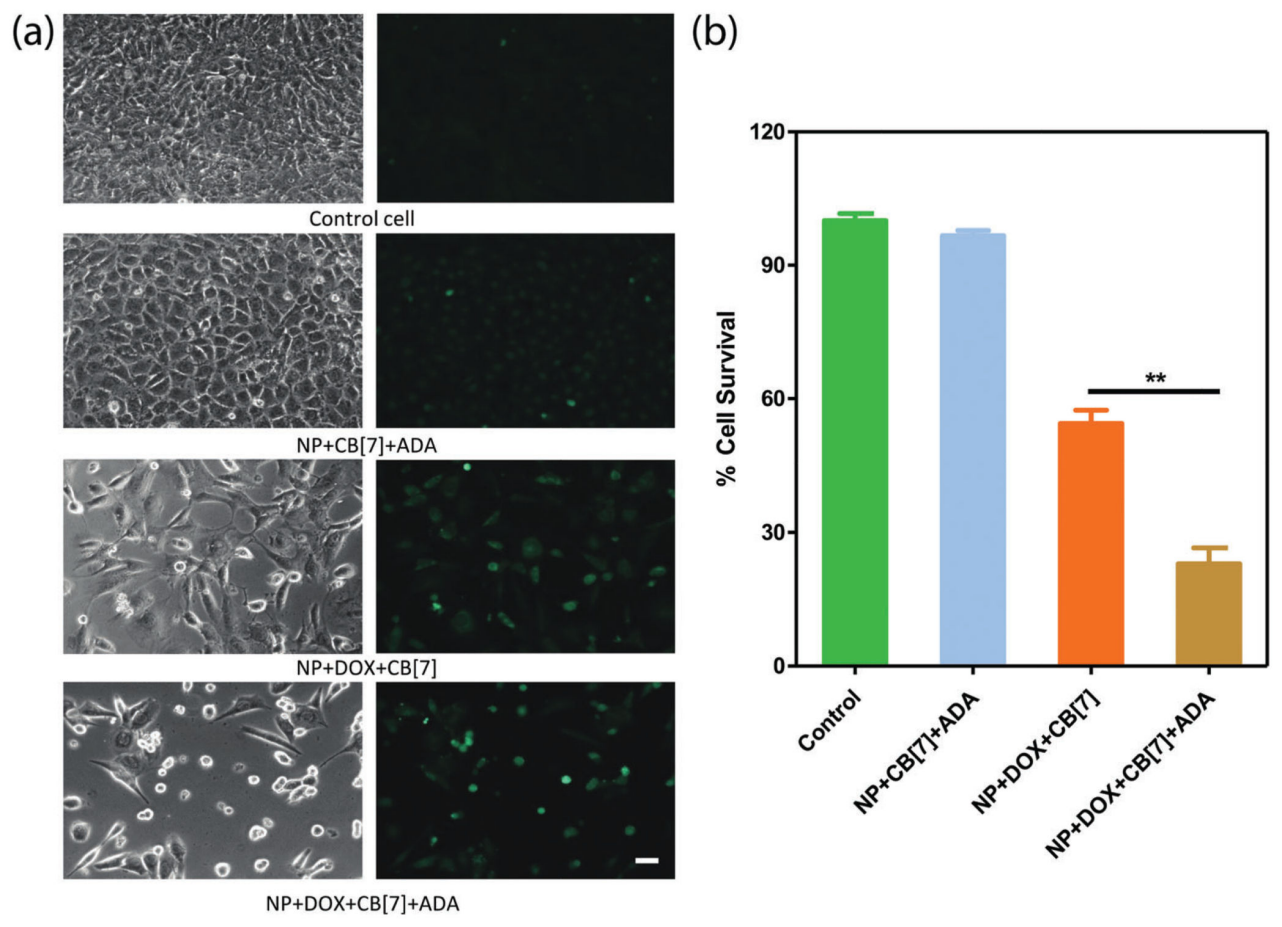
Comparison of cytotoxicity possessed by release of DOX from the AuNP assembly. SVECs were treated with a variety of conditions, including NP + CB[7] + ADA, NP + DOX + CB[7] and NP + DOX + CB[7] + ADA. (a) Bright field image of the cells and the corresponding Calcein AM stained image. (b) Alamar Blue cell viability assay showing the cytotoxicity effect under various conditions. The studies were performed in triplicate and the error bars represent standard error of the mean. ** *p* ≤ 0.01. Scale bar 50 μm.

Finally, we checked the ADA triggered activation *via* cell culture studies. For this study, the Saphenous Vein Endothelial Cell line (SVEC) was treated with the DOX loaded AuNP assembly and triggered with ADA. Additionally, two control experiments were performed to provide support for ADA triggered activation. In one case, we employed the DOX loaded AuNP assembly but performed cell culture studies without the ADA trigger. In another case, we used the AuNP assembly and added the ADA trigger to determine toxicity from the delivery vector. After treating the cells with the control and triggered set, cellular morphology was investigated by acquiring bright field images and Calcein AM staining (Fig. 5a). An Alamar Blue cell viability test was performed to quantitatively assay the cytotoxicity from the different control and triggered sets (Fig. 5b). A significantly increased cell death was observed in the case of the ADA triggered DOX loaded AuNP assembly as compared to the DOX loaded AuNP assembly itself, indicating enhanced drug release upon triggered activation. We also observed that the amount of cell death could be controlled by the amount of given trigger (*i.e.* ADA), indicating the ability to control the drug dosing *via* the given trigger amount (Fig. S19 and S20, ESI†). Additionally, we did not observe any cytotoxicity when cells were treated with only assembly (without DOX) and triggered with ADA, indicating negligible toxicity either from AuNP assemblies or from the ADA trigger (Fig. 5). Overall these results establish that the supramolecular assemblies provide a non-toxic platform that effectively controls the payload release upon exposure to a high-affinity competitive guest.

## Conclusions

In conclusion, we demonstrated a new strategy for controlling drug release rate through the use of competitive interactions of orthogonally presented guest molecules. Triggered release by small chemical stimuli is an important advancement as they could be externally regulated while having the abilities to reach and activate delivery systems in deep tissue. Our system has the capability of easy encapsulation of drug molecules as well as various macromolecular therapeutic molecules in its active and functional state without any chemical modification. Due to ease of fabrication and versatility, we envision that dynamic host–guest nanoparticle assembly directed by a small molecule trigger will play an important role in further developing stimuli responsive drug delivery systems. In addition, we expect that delivery based on reversible supramolecular interaction will pave the way for developing a new strategy for the refilling of implantable drug depots.^35^ Translating this system for *in vivo* delivery is planned and will be reported in due course.

## Supplementary Material

† Electronic supplementary information (ESI) available. See DOI: 10.1039/c8tb01596a

Fig.
