# Supplementary material for: Reversible encapsulations and stimuli-responsive biological delivery from a dynamically assembled cucurbit[7]uril host and nanoparticle guest scaffold†
Source: J Mater Chem B. Author manuscript; Available in PMC 2020 Mar 27. (PMC7100906; doi:10.1039/c8tb01596a)
Supplement: Fig. [file EMS85944-supplement-Fig_.pdf]

Electronic Supplementary Material (ESI) for Journal of Materials Chemistry B.
This journal is © The Royal Society of Chemistry 2018

Electronic Supplementary Information

Reversible Encapsulations and Stimuli-Responsive Biological Delivery from Dynamically Assembled Cucurbit[7]uril Host and Nanoparticle Guest Scaffold

Santu Sinha[1], Nilanjana Das Saha[1,2], Ranjan Sasmal[1], Divyesh Joshi[3], Soumya Chandrasekhar[1,2], Monica Swetha Bosco[1,2], Sarit S. Agasti[1,2,4*]

[1]New Chemistry Unit, Jawaharlal Nehru Centre for Advanced Scientific Research (JNCASR), Bangalore, Karnataka 560064, India.

[2]Chemistry & Physics of Materials Unit, Jawaharlal Nehru Centre for Advanced Scientific Research (JNCASR), Bangalore, Karnataka 560064, India.

[3]Molecular Biology and Genetics Unit, Jawaharlal Nehru Centre for Advanced Scientific Research (JNCASR), Bangalore, Karnataka 560064, India.

[4]School of Advanced Materials" (SAMat), Jawaharlal Nehru Centre for Advanced Scientific Research (JNCASR), Bangalore, Karnataka 560064, India.

*Correspondence e-mail: sagasti@jncasr.ac.in

Synthesis of 5 nm dodecanethiol capped gold nanoparticle

In a round bottom flask, 100 mg of gold (III) chloride trihydrate was dissolved in 50 ml of deionized water with vigorous stirring. 267 mg of tetraoctylammonium bromide (TOAB) in 50 ml toluene was added to the gold salt solution followed by the addition of 54 μl of

dodecanethiol. This two-phase mixture was then stirred for 10 min. To this, 93 mg of $NaBH_4$ in 5 ml cold deionized water, was added quickly and the solution was stirred vigorously for 5 h. The toluene layer was collected and evaporated in a rotary evaporator ensuring that minimum amount of toluene (~5 ml) was retained. The remaining toluene was then transferred to a 25 ml round bottom flask and evaporated to complete dryness and kept in vacuum for 6 h, after which a black layer was formed at the walls of the flask. This crude product was then heated using a heating bath under nitrogen atmosphere. The temperature was increased at the rate of 10 °C every 5 min and maintained at 150 °C for 30 min. The product was allowed to cool to room temperature and was then dissolved in 500 ml methanol. The solution was kept in the freezer (−20 °C) overnight to allow the gold colloids to precipitate and to separate the excess dodecanethiol and TOAB. Methanol was removed by decanting and the nanoparticles were obtained from the precipitate. Finally, the precipitate was dissolved in 5 ml toluene and stored at 4 °C.[1,2]

Synthesis of 2 nm gold nanoparticle (AuNP)

2 nm AuNP was sythesized according to the protocol in reference 3.

Transmission electron microscopic (TEM)characterization of 5 nm dodecanethiol capped AuNP

30 nM of 5 nm dodecanethiol capped NP in toluene was drop casted onto the TEM grid and dried for 24 h in vacuumprior to imaging. TEM images were recorded on the JEOL JEM-3010 instrument operating at 300 kV.

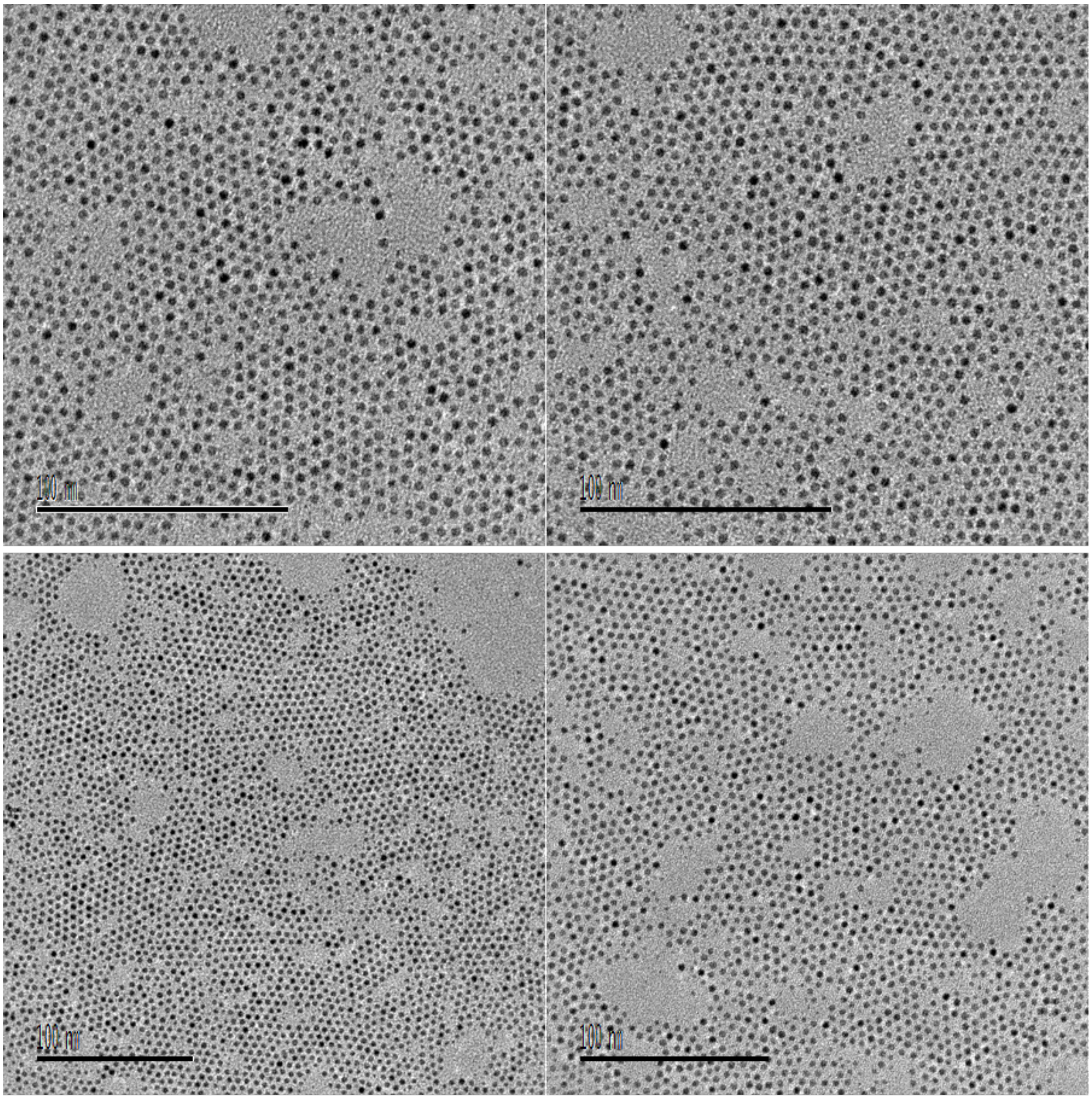

Fig. S1: TEM images of dodecanethiol capped AuNP having average size of ~5 nm.

Post functionalization of 5 nm AuNPs

Benzyl group capped AuNP was synthesized following a previously reported protocol.[4] Briefly, 10 mg of 5 nm dodecanethiol-capped AuNPs were dissolved in 2 ml of dry dichloromethane and purged with nitrogen gas. 50 mg of SH-C_{11}-TEG-NMe_2Bn ligand was dissolved in 2 ml of dry dichloromethane and purged with nitrogen gas.The thiolated ligand

solution was mixed with the AuNP solution and stirred for 48 h. The solvent was thenremoved using the rotary evaporator.In order to remove the excess thiol ligands, AuNPs were washed five-times with hexane (5 ml). After washing, the vial was kept open for complete drying. The AuNP was then dissolved in 1 ml Milli-Q water and dialysedfor 24 h.

Structure of post functionalized benzyl nanoparticle(Benzyl-AuNP)

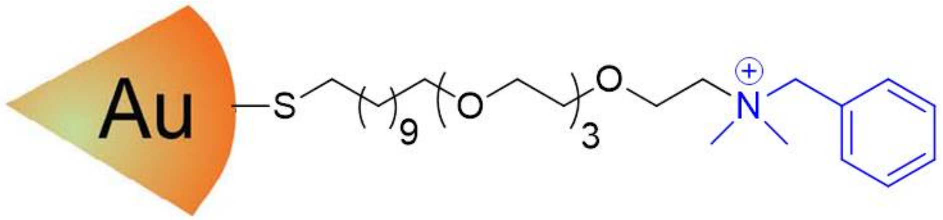

Fig. S2: Structure of Benzyl-AuNP. The nanoparticle is functionalized with a dimethylbenzylamine group through a linker of HS-C_{11}-TEG. The dimethylbenzylamine group is a guest molecule tocucurbit[7]uril (CB[7]), which is the host moiety. Ligand synthesis for the AuNP was done by following the earlier published report.[4]

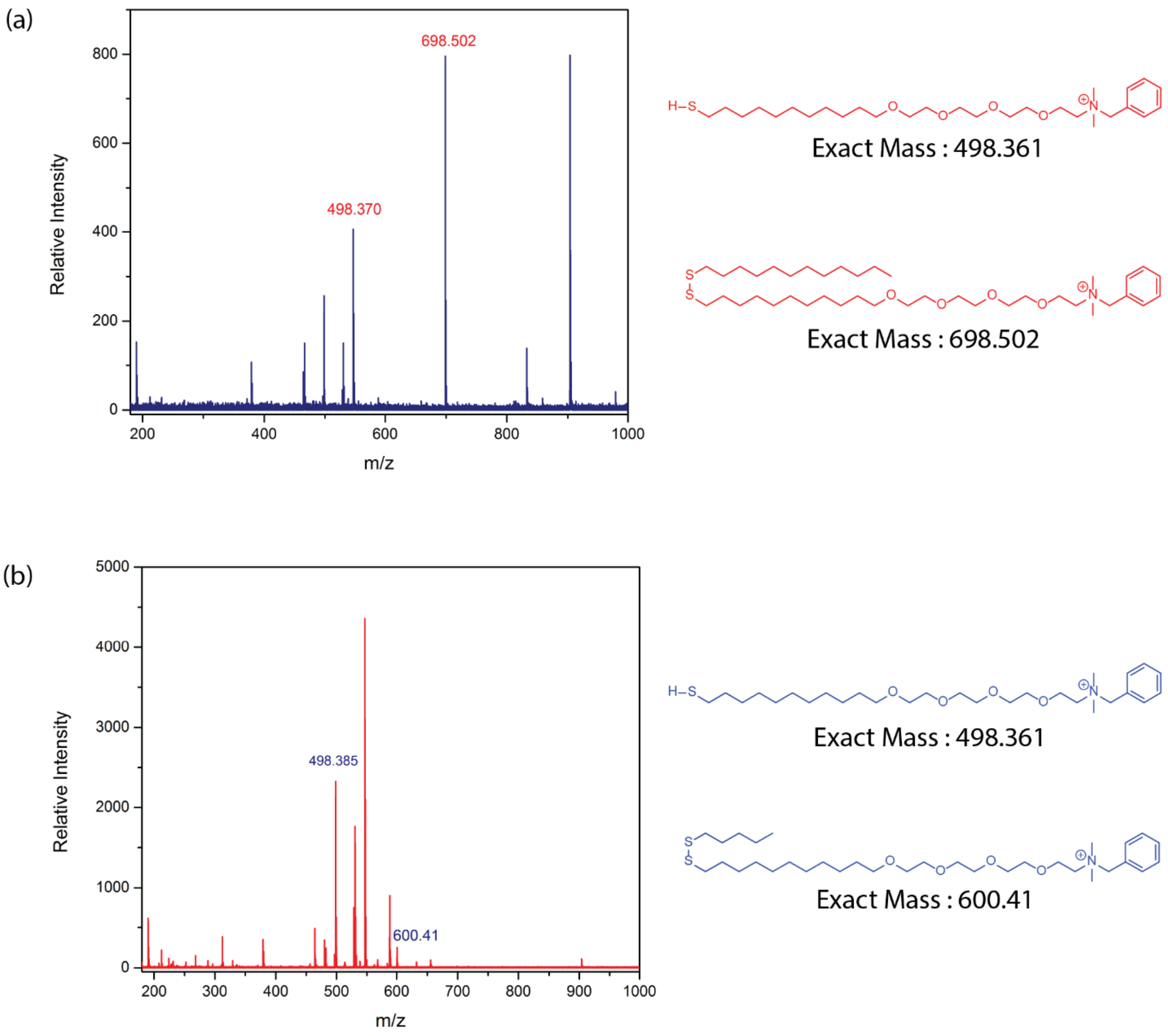

Fig. S3: Mass spectrometric (MALDI) characterization of ligand composition of AuNPs (a) for 2 nm Benzyl-AuNP, (b) for 5 nm Benzyl-AuNP.

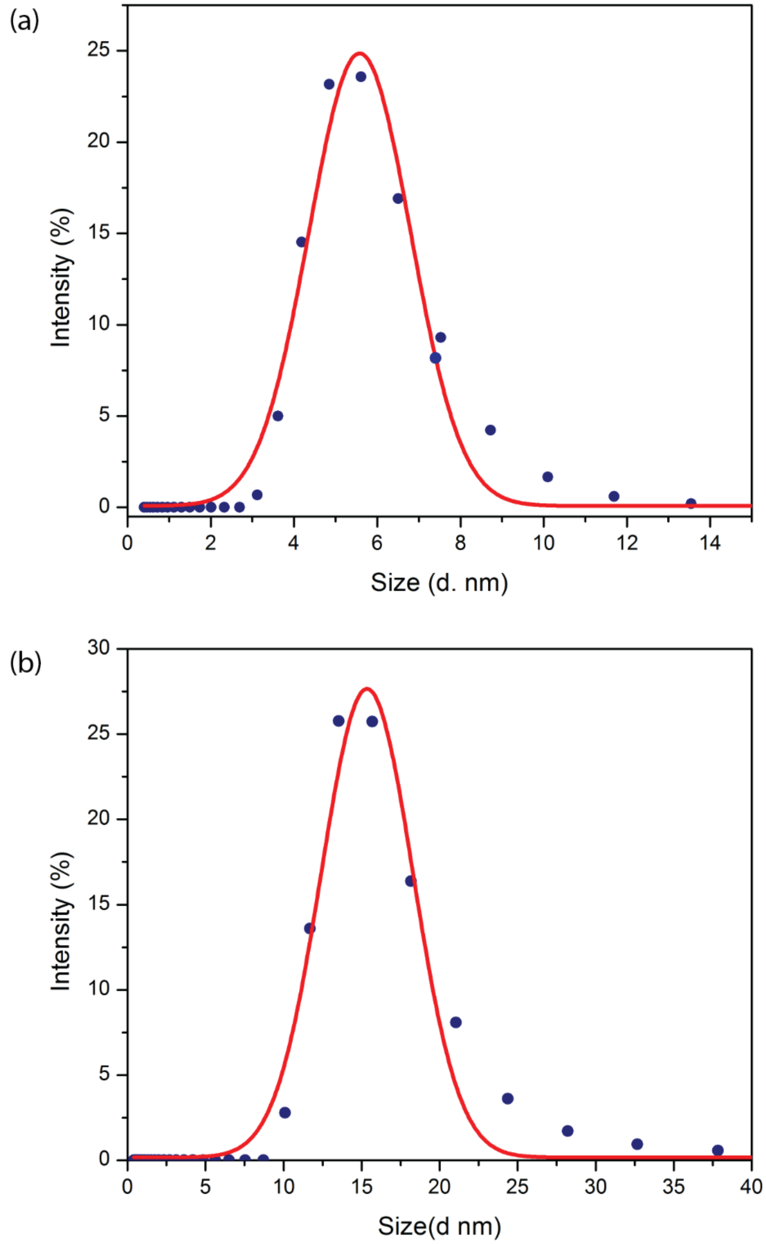

Fig. S4: DLS profiles of the AuNPs (a) 2 nm Benzyl-AuNP and (b) 5 nm Benzyl-AuNP. The concentrations used in the DLS experiments were 200 nM for 2 nm and 30 nM for 5 nm AuNP.

Visual demonstration of AuNP assembly

To 400 μl of 0.2 μM AuNP (5 nm) solution, 24 μl of CB[7] solution (10 mM) was added in order to form the nanoparticle assembly. The assembled nanoparticles settled down at the bottom of the cuvette. An image was acquired after centrifugation, to demonstrate the nanoparticle assembly process. 24 μl of ADA solution (10 mM) was then added for the disassembly process to occur.

Reversibility of surface plasmon resonance of AuNPs

The reversible plasmonic change of AuNP with sequential addition of CB[7] and ADA was studied by recording the UV spectra of the assembly and disassembly process. 400 μl of 5 nm AuNP (37 nM) was taken in the cuvette, followed by the addition of 4.44 μl of 2 mM CB[7] in order to trigger the assembly formation and then the UV spectra was recorded. 2μl of 4.8 mM of ADA was then added to disassemble the AuNP assembly and the corresponding UV spectra was also recorded. For consecutive cycles, the same amount of CB[7] and ADA was added to the solution in an alternative fashion to analyze the surface plasmon resonance of the AuNPs.

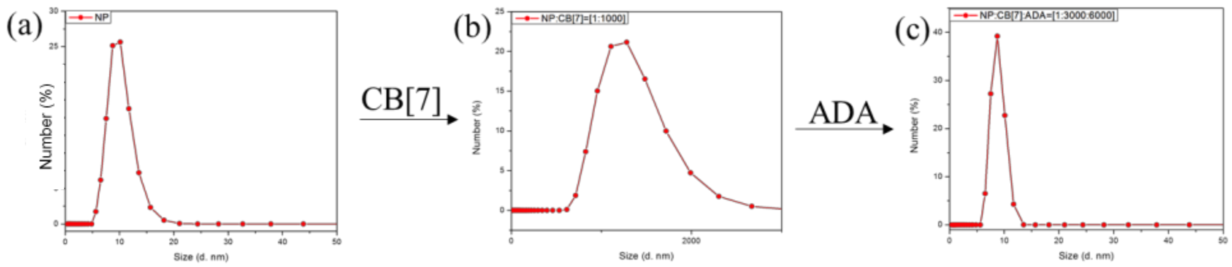

Fig. S5: Reversible nature of AuNP assembly. (a) DLS profile of AuNP solution (b) DLS profile of AuNP-CB[7] assembly (c) DLS profile of AuNP-CB[7] assembly after adding ADA.

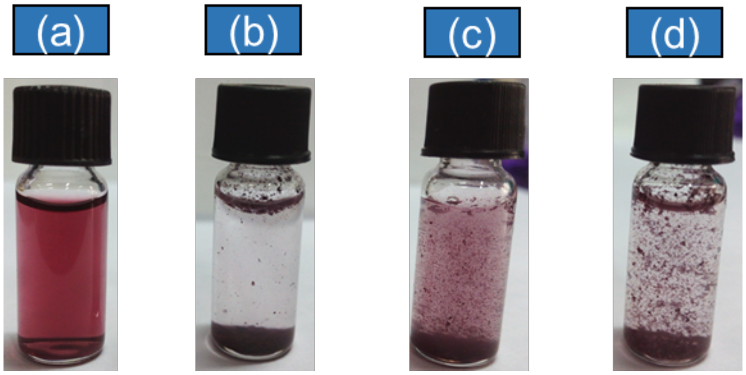

Fig. S6: Images of (a) AuNP solution (b) AuNP+CB[7] (c) heating of AuNP+CB[7] at 50 ºC for 30 min, (d) sonication of AuNP+CB[7] for 5 min (post-heating at 50 ºC) .

Sample preparation for TEM of 5 nm nanoparticle (NP) – CB[7]assembly

To facilitate assembly formation, 4.8 µl of 6 mM CB[7] solution was added to 200 µL of 5 nm benzyl AuNP (30 nM) [C_{AuNP}:$C_{CB[7]}$ =(1:4800)] and allowed to react for 5 min. This solution was then drop-casted onto the TEM grid and dried in vacuum until visualization.

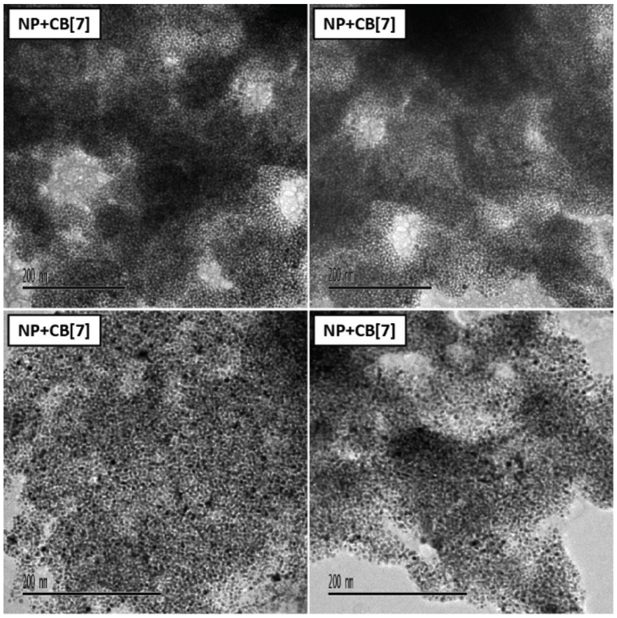

Fig. S7: TEM images of 5 nm Benzyl-AuNP assembly mediated by CB[7].

Sample preparation for AFM

Mica (grade V2, Ted Pella, Inc.) was used as a substrate for the study. Mica layers were peeled off prior to sample deposition to expose a uniform, fresh surface. AuNP and CB[7] were mixed together in appropriate ratios and incubated for 5 min at room temperature. This was then drop casted onto the “virgin” mica surface and incubated for 5 min. Excess, unbound solution was wicked off by washing in Milli-Q water. The surface was then dried using a pure stream of nitrogen gas and stored in vacuum until visualization. The sample was imaged using a Bruker Dimension Icon AFM, in air, under the peak force tapping mode.

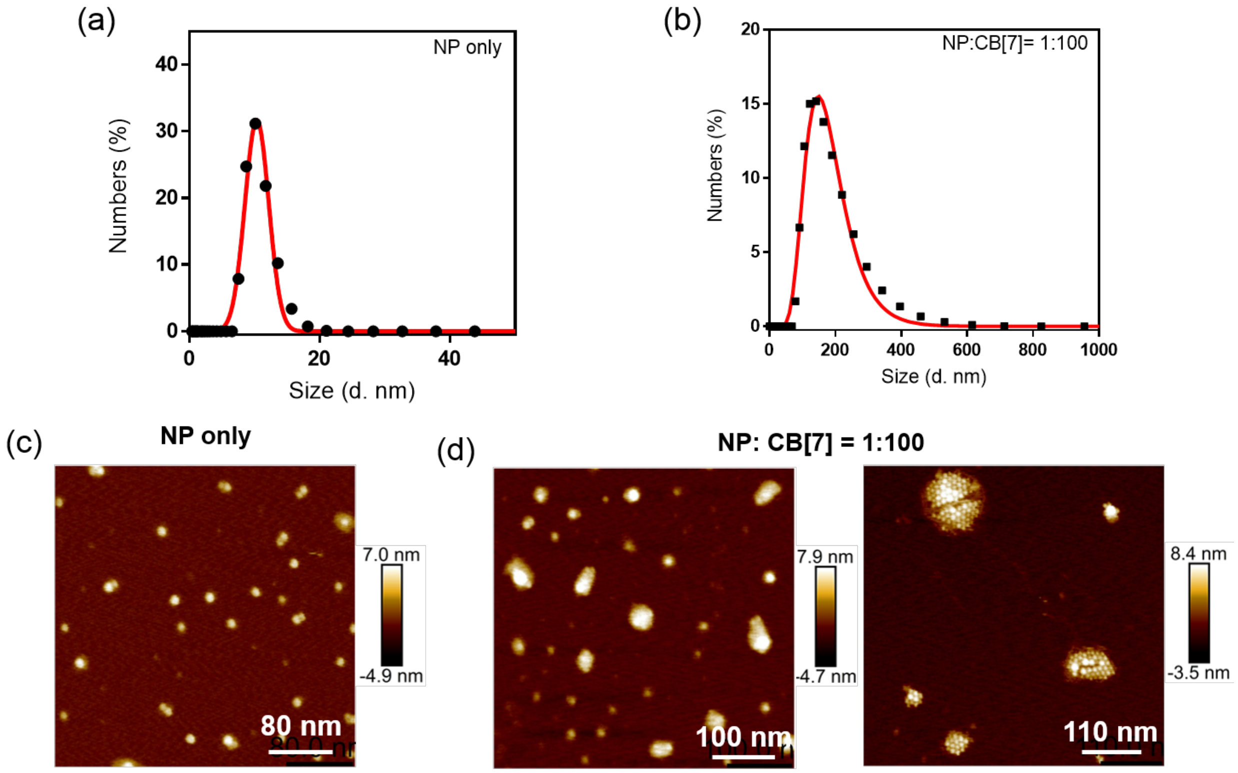

Fig. S8: DLS and AFM data of bare and assembled AuNPs. (a) DLS of bare AuNP showing diameter of around 10 nm, (b) DLS of AuNP assembly with NP:CB[7] ratio of 1:100. It reveals the diameter of AuNP assembly to be around 148.3 nm. (c and d) The respective AFM image of bare AuNP and AuNP assembly with NP:CB[7] ratio of 1:100.

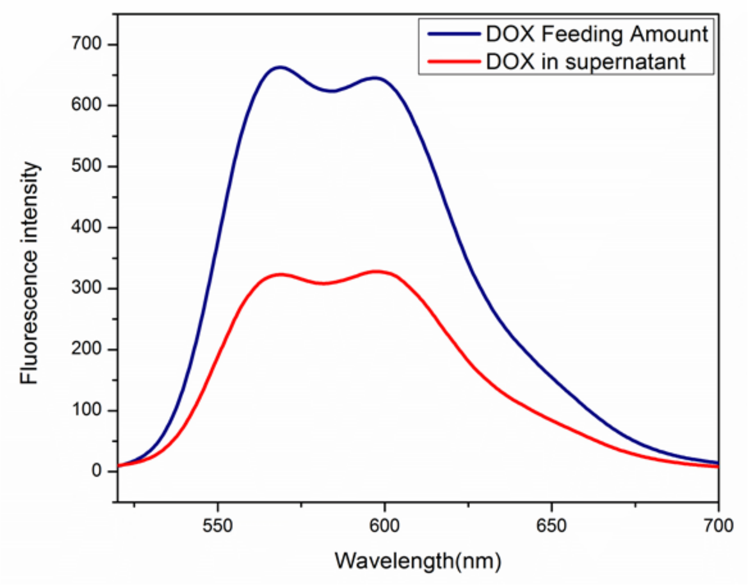

Fig. S9: Percentage of DOX encapsulation during assembly formation of 5 nm Benzyl-AuNP. The amount of DOX encapsulation is found to be 51%. Ratio of different components in the assembly are AuNP:DOX=1:10 and AuNP:CB[7]=1:3000.

Release of Camptothecin (CPT) from 5 nm AuNP assembly

Release of CPT was studied by encapsulating CPT in 5 nm AuNP assembly. To 400 µl of 0.2 µM 5 nm benzyl-AuNP solution, 1 µl of 0.6 mM CPT solution was added and then 12 µl of 20 mM CB[7] solution was added to the mixture. The ratio of the components during assembly formation was C_{NP}:C_{CPT}=1:7.5, C_{NP}:$C_{CB[7]}$=1:3000. After 1 minute incubation, the mixture was centrifuged for 1 minute for the assembly to settle down. The supernatant was removed very carefully without disturbing the assembly. Then 400 µl of PBS solution was added very slowly to avoid dispersionof the assembly in water and thereafter fluorescence was monitored over time by excitation at 370 nm for CPT. Emission spectra wascollected in the range of 380-520 nm for CPT.

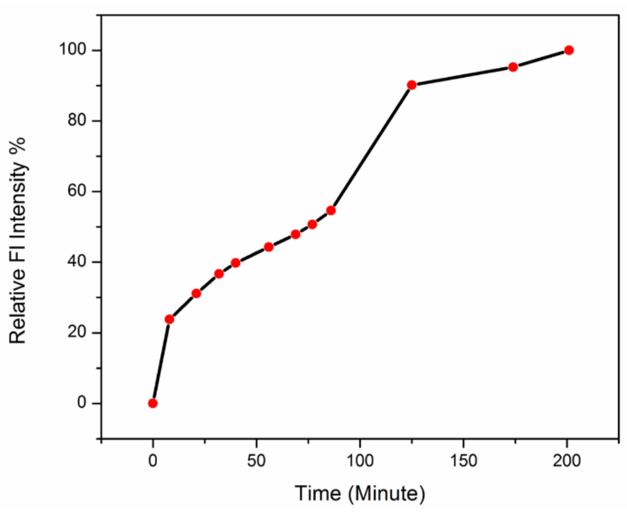

Fig. S10: Extended period of CPT release from 5 nm Benzyl-AuNP assembly over time.

Encapsulation of Doxorubicin (DOX), BSA-FITC, 10 nucleotide(nt)-DNA-Atto-655 conjugate

Encapsulation of drug (DOX), DNA and protein **(**BSA-FITC**)**were donesimultaneously by mixing the three components with AuNP solution and then the assembly was prepared by adding CB[7]. In 2000 µlof PBS solution in a 3.5 ml fluorescence cuvette, 7.5 µl of 0.67 mM DOX, 2 µl of 100 µM BSA-FITC and 2 µl of 100 µM 10 nucleotide (nt)-DNA-Atto-655 conjugate solutions were addedand fluorescence of all the three compounds were measured. To 2000 µl of 0.2 µM 5 nm benzyl-AuNP solution, all the three compounds were added in the same amount in the presence of CB[7] for assembly formation. 200 µl of 6 mM CB[7] soln was added to the mixture and left undisturbed for 10 min for the assembly to settle down at the bottom.The ratio of the components in the solution during assembly formation were C_{NP}:C_{DOX}=1:12.5, C_{NP}:$C_{DNA\text{-}Atto}$

$=1:0.5$ and $C_{NP}:C_{BSA\text{-}FITC} = 1:0.5$, $C_{NP}:C_{CB[7]} = 1:3000$. After the assembly settled at the bottom, fluorescence measurement of the supernatant was performed. The fluorescence measurement was carried out by exciting all the fluorophores separately, λ_{Ex}(DOX)=480 nm, λ_{Ex}(FITC)=495 nm, λ_{Ex}(Atto-655)=650 nm and the emission was collected in the range of 520-760 nm for DOX, 510-850 nm for FITC, 660-850 nm for Atto-655.

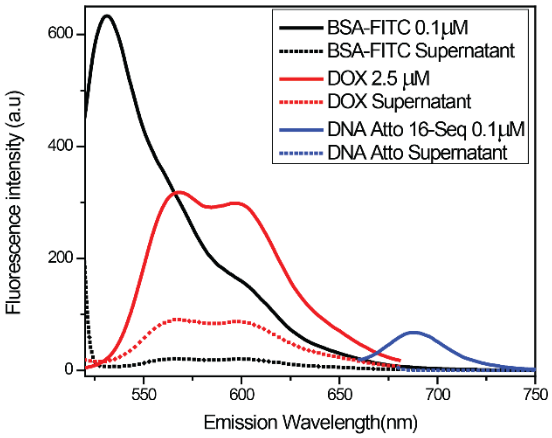

Fig. S11: Simultaneous encapsulation of multiple therapeutics in 5 nm Benzyl-AuNP assembly. Encapsulation of BSA-FITC, DOX and 10-nt-DNA-Atto in the nanoparticle assembly.

Encapsulation of enzyme and catalysis

For a visual demonstration of encapsulation and catalytic activity of an encapsulated enzyme,β-galactosidase was added to the AuNP solution and the assembly was made. To 550 µl of 0.1 µM 5 nm benzyl-AuNP solution, 30 µl of 500 nM β-galactosidase was added and then the assembly was made by adding 9.16 µl of 15 mM CB[7] solution followed by centrifugation and careful removal of the supernatant to avoid any loss of the assembly. Further, the assembly was washed with 100 µl PBS solution twice. 500 µl PBS solution was then added to the assembly and freshly

prepared 20 µl of 5 mM o-nitrophenyl β-galactoside was added and the colour change was observed over time. Multiple cycles of catalysis were performed bythe removal and washing ofthe supernatant from the assemblywith 100 µl PBS, followed by the addition of 500 µl PBS. Same amount of the substrate was added for further catalysis.

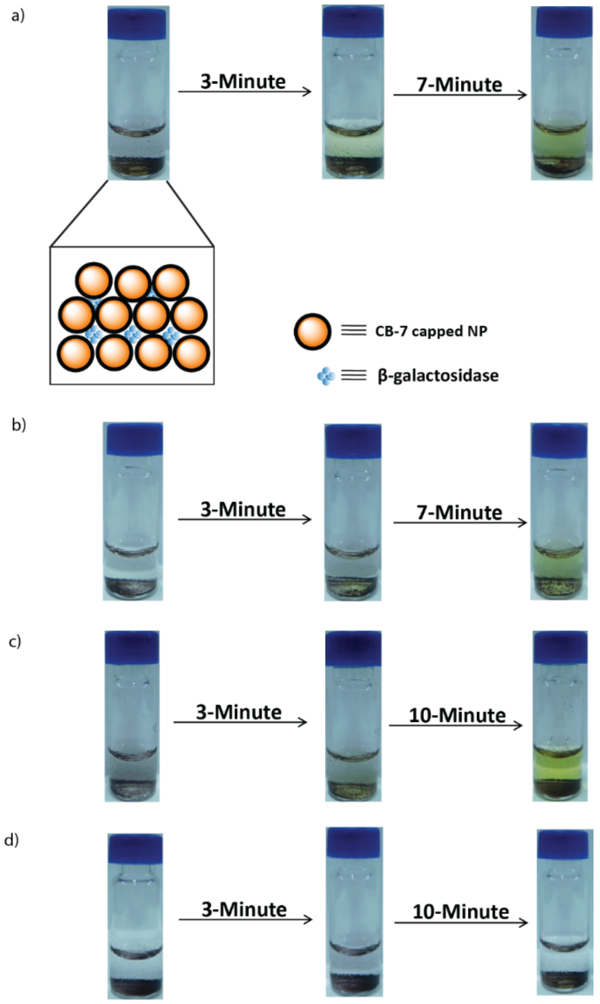

Fig. S12: β-galactosidase encapsulation within the assembly and repetitive activity of the encapsulated enzyme on ortho-nitrophenyl-β-glactoside(ONPG). **a)**Enzymaticcleavage of ONPG by encapsulated enzymein the first cycle, **b)** Enzymatic cleavage of ONPG in the second cycle,

c) Enzymatic cleavage of ONPG in the third cycle. **d)** Control-indicating the assembly not containing enzyme encapsulationand therefore no color change wasobserved upon addition of substrate. In all cases the assembly settled at the bottom of the vial.

The procedure of implant fabrication

For the study of DOX release kinetics from NP assembly, an implant of cylindrical shape was made by cutting pipette tips and one cross-section of the tip was covered with nitrocellulose membrane (pore size 0.2 μm). First, the pipette tips were cut into two piecesand upper part was used for implant fabrication. The smooth outer part was heated slightly and pressed on top of the nitrocellulose membrane in hot condition so that the tip adheres to the membrane. The membrane was used to avoid transfer of assembled materials to the medium (as NPs were seen to create problems in fluorescence measurements) and allow only drug molecules to diffuse from the assembly to the media. For the drug release and cell culture study, membrane attached parts of the implant were placed on individual wells of a 24 well plate using double sided tape. Figure S10 illustrates the method of preparation of an implant.

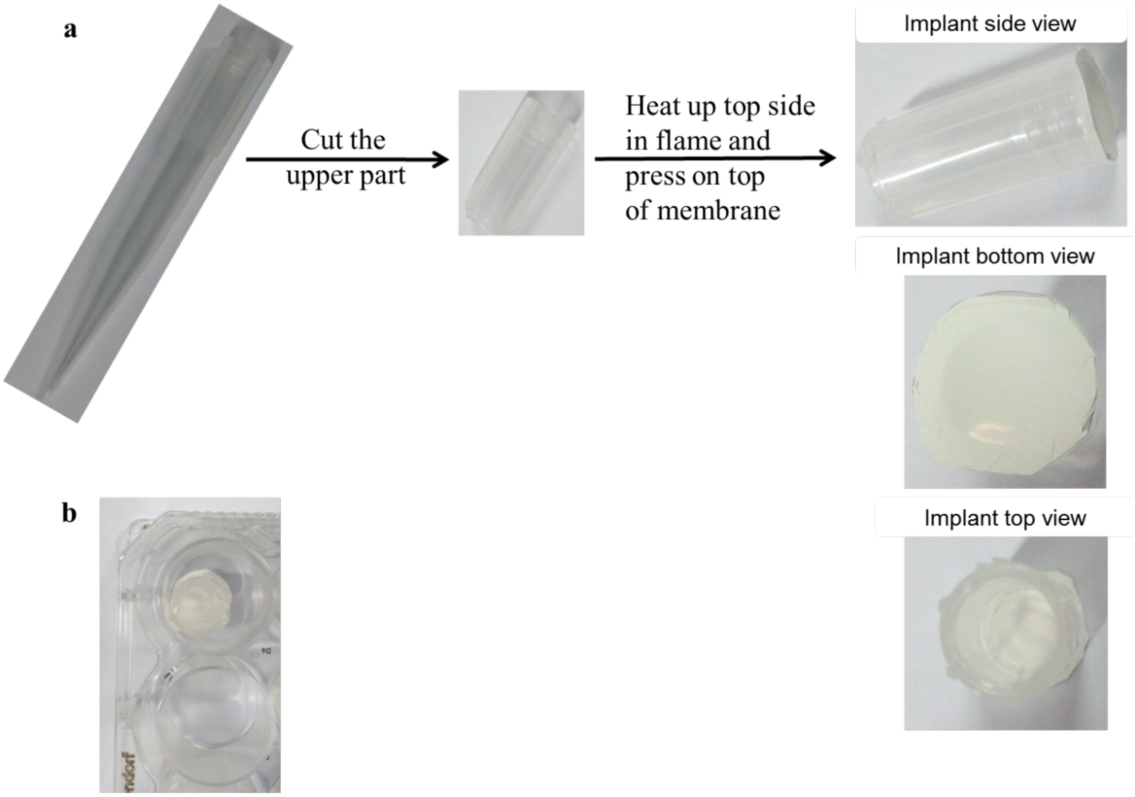

Fig. S13: Implant fabrication process used for drug release and cell culture study. **a)** Images offabrication of the implant from 1 ml pipette tips.**b)** Picture of implant placed inside the well of a 24 well plate.

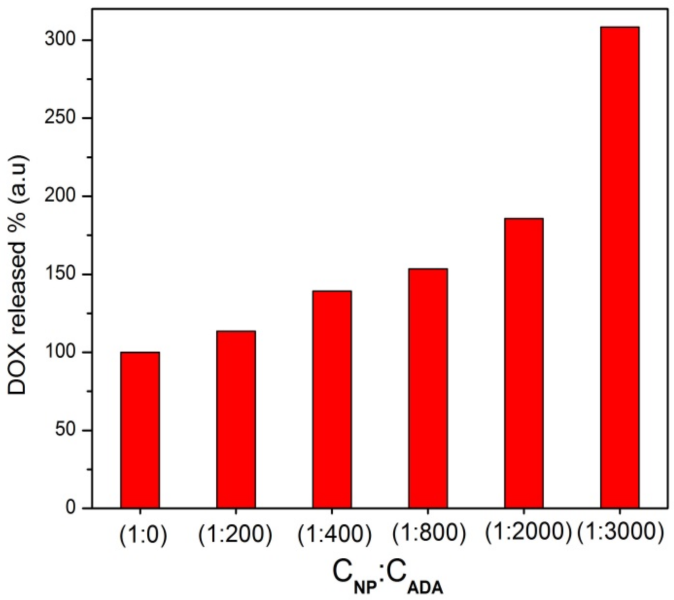

Fig. S14: Drug release from 5 nm AuNP assembly by different ratios of ADA.

ADA triggered DOX release study

In a 500 µl centrifuge tube, 6 µl of 25 µM 5 nm benzyl-AuNP solution taken and to that 18 µl of 2.5 mM DOX was added,this was followed by the addition of 22.5 µl of 20 mM CB[7] to make the DOX encapsulated assembly. The ratioof the components used in assembly formation was C_{NP}:C_{DOX}=1:300 and C_{NP}:$C_{CB[7]=}$1:3000. The mixture was centrifuged and the supernatant was removed. The assembly was then washed with 50 µl of PBS solution and transferred inside the implantwith 100 µl of PBS solution (dipped into 1 ml PBS solution in a 24 well plate). Two sets of asembly were made, one for normal diffusion based release and another for ADA triggered release. The experiments were performed in triplicate. For the triggered set, 22.5 µl of 20 mM ADA was added inside the implant, and the ratio of stimulant was $C_{CB[7]}$:C_{ADA}=1:1.Fluorescence measurement was performed at different time points in a microplate reader using Corning make black 96-well plate by taking 25 µl solution from the well plate and diluting it to 50 µl. Excitation for the DOX was set at 480 nm and emission was collected at 570 nm.

Assembly preparation for BSA coated DOX release

Assembly was prepared as mentioned previously for ADA triggered DOX release.For coating with BSA, 1 µl of 22.5 µM BSA solution was added to the preformed assembly and transformed inside the implant in the same way as mentioned earlier. For the triggered set implant, 7.5 µl of 20 mM ADA was added inside the implant. Fluorescence measurements were done by taking10 µl of solutionand diluting it to 50 µl. These experiments were performed in triplicate.

***In situ* DOX release study from 5 nm AuNP assembly**

In a 0.5 ml microcentrifuge tube 10 µl of 7.5 µM 5 nm benzyl nanoparticle solution and 9.1 µl of 2.5 mM DOX solution was mixed together followed by the addition of 7.6 µl of 20 mM CB[7] solution to make the DOX loaded NP assembly. The mixture was centrifuged and the supernatant was removed. The assembly was then washed with 50 µl PBS solution carefully to avoid the loss of particles. This was followed by the addition of 100 µl PBS into the tube from which 5 µl solution was taken out at every time point for measurements. At the 20 min time point 7.6 µl of 20 mM ADA solution was added into the tube for trigger action. Ratio of the component used for this study was NP: DOX: CB[7] = 1:300:2000 and CB[7]:ADA=1:0.125.

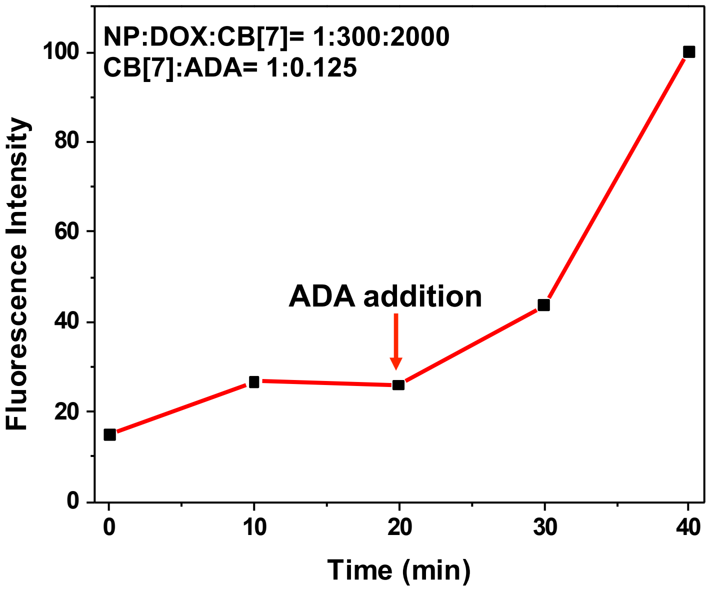

Fig. S15: *In situ* triggered release study of doxorubicin using ADA as external trigger. NP assembly was made using NP:DOX:CB[7] ratio of 1:300:2000. Triggered release experiment has been carried out after addition of ADA at 20 min time point with CB[7]:ADA ratio of 1:0.125.

***In situ* DOX release study from 2 nm AuNP assembly**

In a 0.5 ml microcentrifuge tube 40 µl of 25 µM benzyl nanoparticle solution (2 nm) and10 µl of 2.5 mM DOX solution was mixed together followed by the addition of 5 µl of 20 mM CB[7] to make the DOX loaded NP assembly. The mixture was centrifuged and the supernatant was removed. The assembly was then washed with 50 µl PBS solution carefully to avoid the loss of particles.This was followed by the addition of 100 µl PBS into the tube from which 5 µl solution was taken out at every time point for measurements. At the 20-minute time point 5 µl of 20 mM ADA solution was added into the tube for trigger action. The ratio of the components used for this study was NP: DOX: CB[7] = 1:25:100 and CB[7]:ADA=1:1**.**

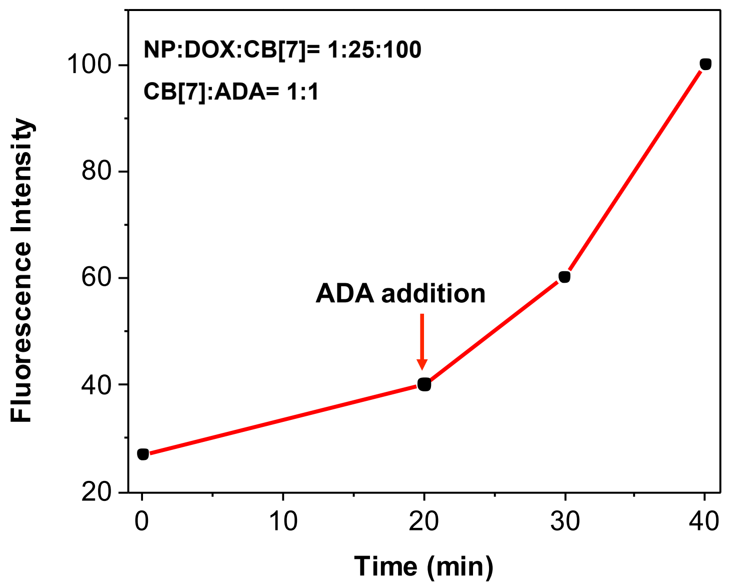

Fig. S16:*In situ* triggered release study of doxorubicin using ADA as external trigger. NP (2 nm) assembly was made using NP:DOX:CB[7] ratio of 1:25:100. Triggered release experiment has been carried out after addition of ADA at 20 min timepoint with CB[7]:ADA ratio of 1:1.

SVEC cell culture and imaging study

Saphenous vein endothelial cell line (SVEC) was cultured in DMEM containing 10% FBS (Gibco-BRL, USA) and 2 mM Glutamax (Invitrogen, Carlsbad, USA). Cells were cultured in 24-well plate for 24 h to 60% confluence before starting the drug treatment. Drug treatments were performed using fabricated implants as mentioned earlier. Cells were treated with assembly for a period of 24 h and then washed twice in 1X Phosphate-Buffered Saline (PBS). Thereafter, Calcein dye (Invitrogen, Carlsbad, USA) was added at a concentration of 5 µM and cells were incubated at 37˚C in CO_2 incubator for an additional time of 30 min. Cells were washed twice in 1X PBS and imaged in complete phenol red free cell culture medium.

Brightfield (phase contrast) or fluorescence images were captured with an inverted fluorescence microscope (IX70, Olympus)using 10X plan objective equipped with a cooled charge coupled

device (CCD) camera (CoolSNAP; Roper Scientific, Inc), keeping exposure times equal for each fluorescence channel across drug treatments.

Method of assembly preparation and light triggered DOX release study

For the light triggered DOX release study, the NP assembly was preparedin the ratio of C_{NP}:C_{DOX}=1:300, C_{NP}:C_{ADAPC}=1:6000, C_{NP}:$C_{CB[7]=}$1:3000. At first 37.1 µl of 4.04 µM solution of 5 nm benzyl-AuNP solution was taken in a 500 µl centrifuge tube, to which 18 µl of 2.5 mM DOX and 4.96 µl of 181 mM ADAPC solution were added and mixed properly. To make the assembly, 22.5 µl of 20 mM CB[7] was added to the mixture. After instantaneous assembly formation, the tube was centrifuged and the supernatant was removed followed by further washing with 50 µl PBS solution. The resultant assembly was then transferred inside the implant (which was dipped into 1 ml PBS solution in a 24 well plate) using 100 µl PBS. For trigger action, the triggered set was irradiated with UV light (50 mW) for 60 seconds from the top of the implant. Fluorescence measurement was performed in a microplate reader using Corning make black 96-well plate by taking 25 µl solution from the well plate and diluting it to 50 µl. Excitation for the DOX was set at 480 nm and emission was collected at 570 nm.

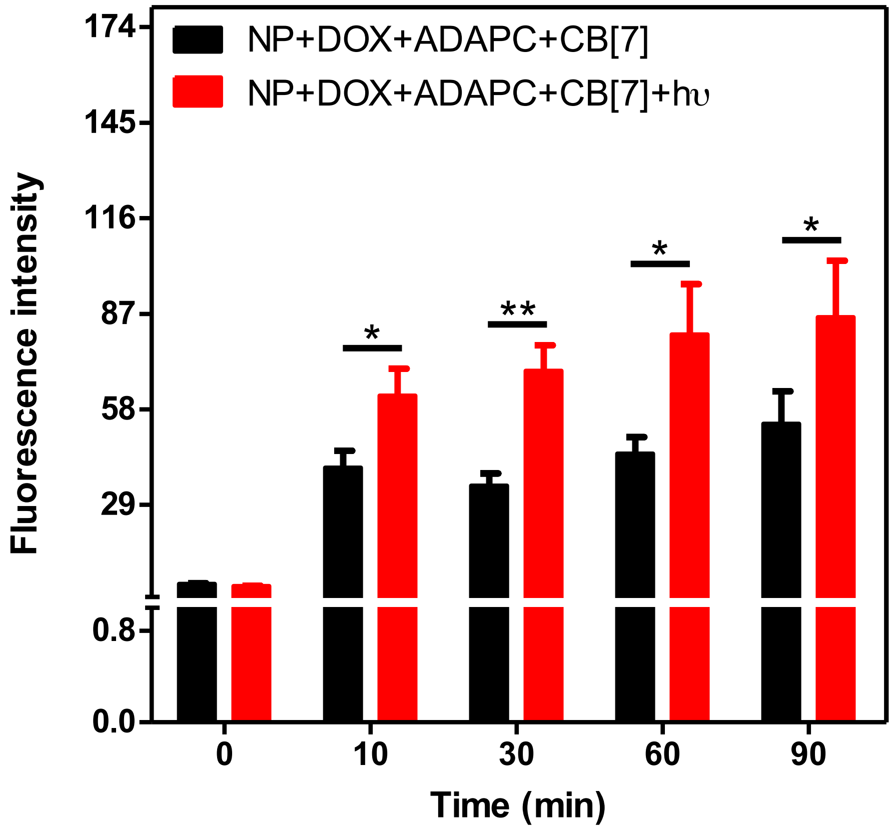

Fig. S17: Light stimulated release of DOX from 5 nm Benzyl-AuNP assembly, controlled and triggered by UV. Ratio of componentNP: DOX=1:300, NP: ADAPC=1:6000, NP: CB[7]=1:3000. The triggered set was irradiated with 365 nm UV of intensity 50 mW for 60 seconds. The studies were performed in triplicate and the error bars represent standard error of the mean. * $p \leq 0.05$, ** $p \leq 0.01$.

Assembly preparation for SVEC cell culture study

43µl of 3.7 µM5 nm Benzyl-AuNP solution and 5µl of 0.8 mM DOX solution was mixed and to that,140 µl of 4.4 mM of CB[7] solution was added to make the assembly. The solution was centrifuged for 1 minute at 50 rpm and the supernatant was removed and washed with 50 µl PBS solution. In case of assembly without DOX, the solution was made in a smilar way (without adding DOX). The assembly was then transferred inside the implant using 100 µl cell culture media and placed inside the well of a 24 well plate as shown in S11 . For the triggered set, the ADA solution(12 mM)of 13.2 µl, 26.4 µl and 39.6 µl was added inside the implant for the cases where ratio of CB[7]:ADA was in the order of 1:0.3, 1:0.6 and 1:1 respectively.

Alamar Blue Assay

SVEC cells were used for the cytotoxicity assay. The cells were cultured in a humidified atmosphere (5% CO_2) at 37°C and grown in Dulbecco's Modified Eagle's Medium (DMEM, high glucose) supplemented with 10% fetal bovine serum (FBS) (Gibco, USA), 2 mM Glutamax (Invitrogen, USA) and 1% antibiotics (100 units/ml penicillin, 100 µg/ml streptomycin and 0.25 µg/ml amphotericin) (Gibco, USA). Prior to experiment, the cells were seeded at ~50,000 cells/1000µl in a 24 well plate. After 24 h, the cells were washed with 1× DPBS. Then implant-containing assembly was dipped into the cell culture media and incubated for 2.5 h. The implant was then removed and cells were kept in the incubator for 24 h. After 24 h, cells were washed and incubated with media containing Alamar Blue solution (90 µl media + 10 µl Alamar Blue stock per well) (as per standard protocol). After ~4 h incubation, the absorbance was measured at 570 nm (experimental wavelength) and 600 nm (reference wavelength) and the data was analyzed by GraphPad Prism.

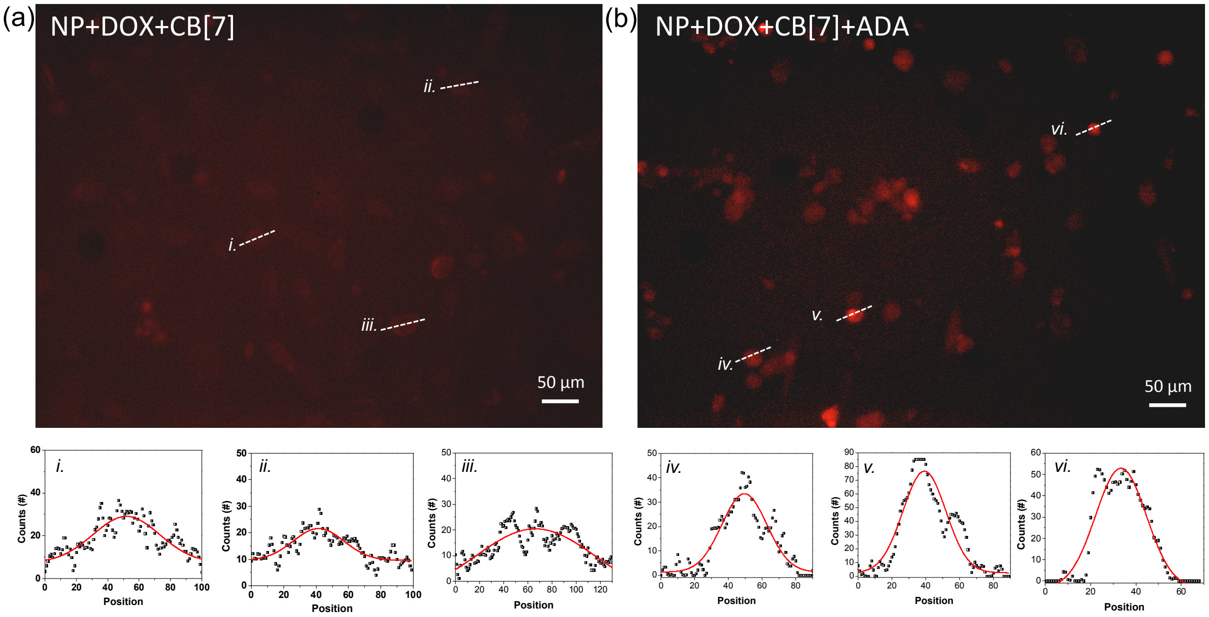

Fig. S18: Fluorescence images of SVEC cell nucleus treated with (a) NP+DOX+CB[7] and (b) with NP+DOX+CB[7]+ADA. Significantly enhanced fluorescence intensity in presence of ADA indicates enhanced amount of DOX delivery upon triggered activation. Intensity profile of fluorescence signal from doxorubicin has been plotted for NP+DOX+CB[7] (i-iii) and for NP+DOX+CB[7]+ADA (iv-vi). Treatment of ADA showed around three fold increase of fluorescence signal from cell. All the fluorescence images are represented with same brightness and contrast window.

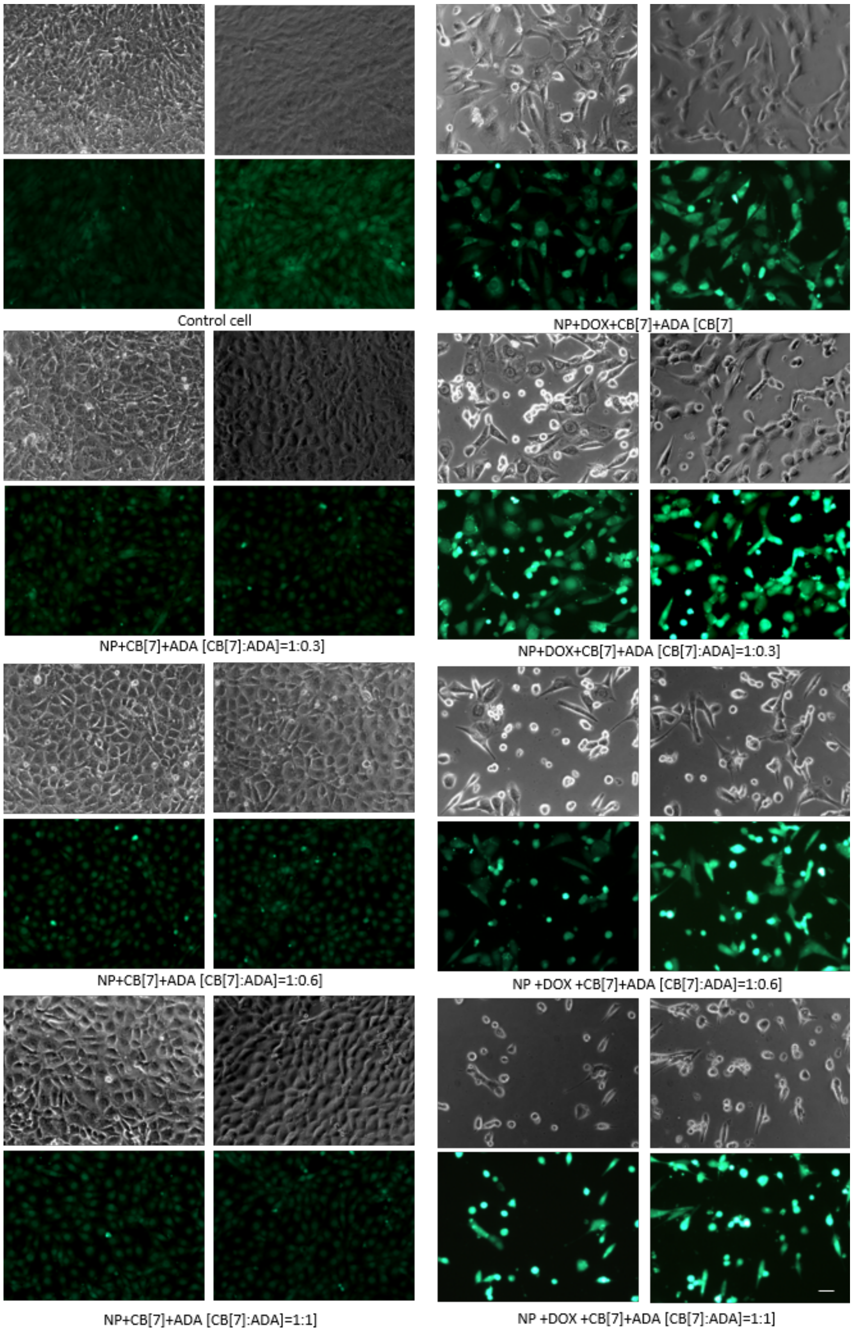

Fig. S19: Brightfield and fluorescence images of SVEC Cell treatedwith NP+CB[7] assembly or NP+DOX+CB[7] and triggered with ADA in all different ratio of ADA. Left side panel shows control cell and cell treated with NP+CB[7] that are triggered with ADA. Right side panel shows

cell treated with NP+DOX+CB[7] and triggered with ADA. For visualiziation cells were treated with Calcein AM. Scale bar 50 μm.

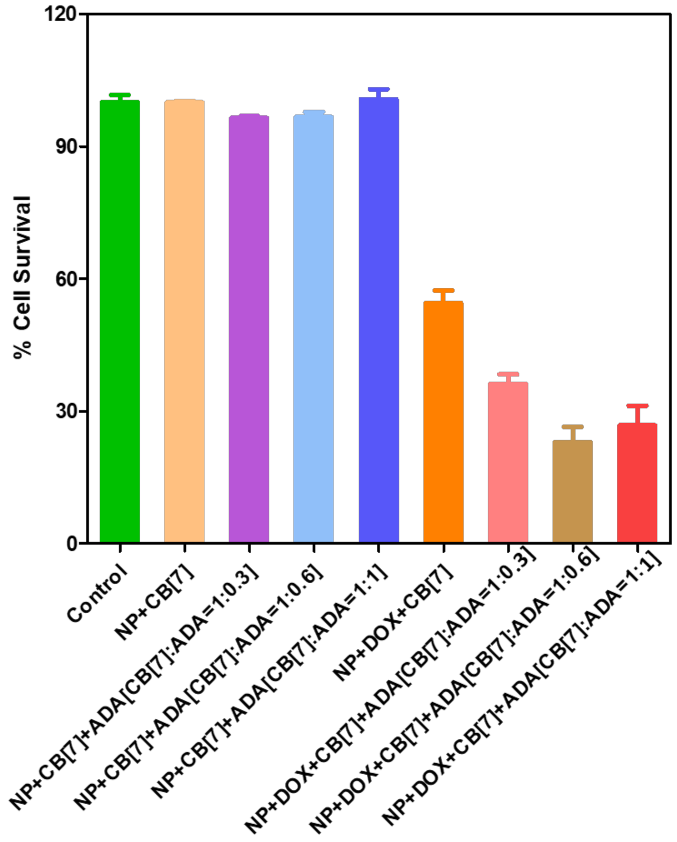

Fig. S20: Cell viability for different conditions provided in Fig. S18. Cell viability was measured by almar blue assay after 24 h.

Fluorescence microscopy imaging in structured illumination method

HeLa cells seeded in 35 mm cell culture dish was treated with DOX loaded AuNP assembly and placed under the microscope for imaging.Fluorescence microscopic images were captured by structured illumination method using an inverted Zeiss ELYRA PS1 microscope.561 nm (200

mW) laser system has been used for excitation. 10% of total laser coming out from objective (40 mW) was used for imaging using a Zeiss oil–immersion objective (alpha Plan–apochromat DIC 63x/1.40 Oil DIC M27, numerical aperture (NA) 1.40 oil). Fluorescence light was spectrally filtered with emission filters (MBS– 561+EF BP 570–650/LP 750 for laser line 561 and imaged using a PCO edge sCMOS camera. Acquired images were processed using Zen 2.0 software and additional softwares have been used for color adjustment (ImageJ).

Synthesis of photocleavable group(PC), PC-NHS and ADAPC

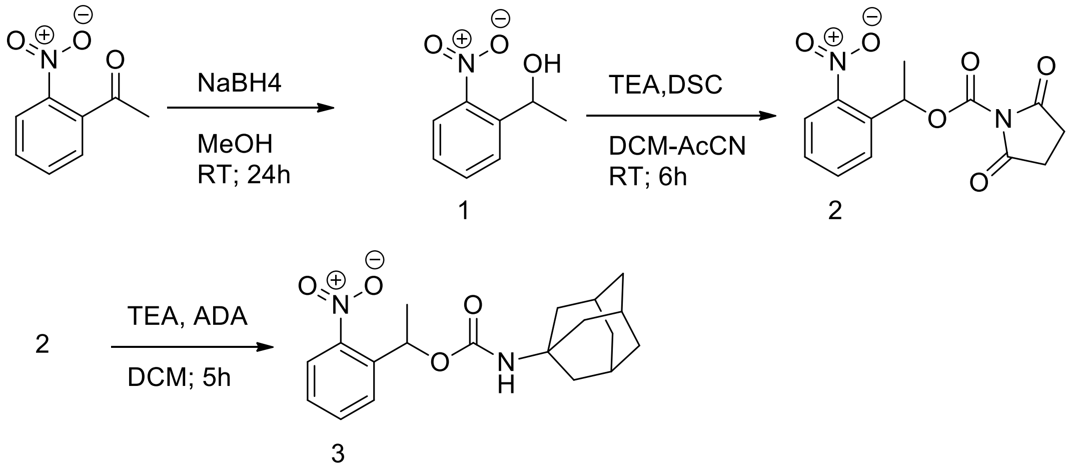

Synthesis of Compound 1 (PC): 1-(2-nitrophenyl) ethanone (400 mg; 2.42 mmol) was dissolved in 10 ml HPLC grade methanol. $NaBH_4$ (366 mg; 9.68 mmol) was slowly added to the solution by keeping the round bottom flask in ice-cold condition; icewas then removedand stirring was carried on for 16 h at room temperature.After completion of the reaction, the solventwas evaporated using a rotary evaporator. Water was added to the reaction mixture and extraction was done using ethyl acetate thrice. The organic layers were combined and dried over Na_2SO_4,followed by solvent evaporation and drying in vacuum to get the product. The yield was

90%.^{1}H NMR ($CDCl_3$, 400 MHz): δ 7.93-7.80 (m, 2H), 7.68-7.62(t, 1H), 7.45-7.39(t, 1H), 5.46-5.38(q, 1H), 2.28(br, 1H), 1.58-1.55(d, 3H).[5]

Synthesis of Compound 2 (PC-NHS): Compound 1 (1.3 g; 7.7 mmol) was dissolved in 10 ml AcCN-DCM (1:1) dry solvent under nitrogen atmosphere.Triethylamine(TEA) (3.24 ml; 23.31 mmol) was then added to the reaction mixture.This was followed by the addition of DSC (5.97 gm; 23.31 mmol)to the reaction mixture which was stirred for 6 h.DCM was then added to the reaction mixture up to a volume of 30 ml. Extraction was done by using a separating funnel with 20 ml 1N HCl. The aqueous layer was removed and again extracted with 20 ml saturated NaCl solution. After removal of the aqeous layer, the DCM layer was passed over anhydrous Na_2SO_4. The solvent was removed by using a rotary evaporator,and the crude reaction mixture was obtained. The mixture was purified by flash chromatography using hexane-ethyl acetate as the solvent system. The yield was 70%. ^{1}H NMR ($CDCl_3$, 400 MHz): δ 8.04-7.99(d, 1H), 7.76-7.71(m, 2H), 7.52-7.47(m, 1H), 6.42-6.36(q, 1H), 2.79(s, 4H), 1.80-1.77(d, 3H).[5]

Synthesis of Compound 3 (ADAPC):ADA (50 mg, 0.33 mmol) was dissolved in 2 ml of dry DCM in 25 ml round bottom flask. TEA (0.134 ml, 0.99 mmol) was then added to the round bottom flask, followed by the addition of compound 2 (101 mg, 0.33 mmol) and was kept under stirring for 5 h. After this, DCM was removed by using the rotary evaporator, and the crude mixture was obtained. The crude mixture was again dissolved in 5 ml DCM and extracted with water in the separating funnel. DCM layer was collected, dried and then purified by flash chromatography. The yield was 50%. ^{1}H NMR ($CDCl_3$, 400 MHz): δ 7.98-7.88(m, 1H), 7.67-7.56(m, 2H), 7.45-7.35(m, 1H), 6.26-6.15(q, 1H), 4.66-4.53(br, 1H), 2.05(m, 3H), 1.93-1.90(d, 3H), 1.69-1.52(m, 12H).

References

1. S. C. Hayden, G. Zhao, K. Saha, R. L. Phillips, X. Li, O. R. Miranda, V. M. Rotello, M. A. El-Sayed, I. Schmidt-Krey and U. H. F. Bunz, *Journal of the American Chemical Society*, 2012, **134**, 6920-6923.

2. T. Teranishi, S. Hasegawa, T. Shimizu and M. Miyake, *Advanced Materials*, 2001, **13**, 1699-1701.

3. D. F. Moyano, B. Duncan and V. M. Rotello, *Methods in molecular biology (Clifton, N.J.)*, 2013, **1025**, 3-8.

4. G. Y. Tonga, Y. Jeong, B. Duncan, T. Mizuhara, R. Mout, R. Das, S. T. Kim, Y.-C. Yeh, B. Yan, S. Hou and V. M. Rotello, *Nature Chemistry*, 2015, **7**, 597.

5. Y. Kikuchi, J. Nakanishi, T. Shimizu, H. Nakayama, S. Inoue, K. Yamaguchi, H. Iwai, Y. Yoshida, Y. Horiike, T. Takarada and M. Maeda, *Langmuir*, 2008, **24**, 13084-13095.
